# Exploring the potential of computer simulation models in drug testing and biomedical research: a systematic review

**DOI:** 10.3389/fphar.2025.1644907

**Published:** 2025-09-24

**Authors:** Rahul Mittal, Alan Ho, Harini Adivikolanu, Muskaan Sawhney, Joana R. N. Lemos, Mannat Mittal, Khemraj Hirani

**Affiliations:** ^1^ Diabetes Research Institute, University of Miami Miller School of Medicine, Miami, FL, United States; ^2^ Division of Endocrinology, Diabetes, and Metabolism, Department of Medicine, University of Miami Miller School of Medicine, Miami, FL, United States

**Keywords:** simulation models, computational modeling, regulatory science, biomedical research, animal alternatives, AI in drug discovery, translational research

## Abstract

**Introduction:**

The growing limitations of animal models in drug testing and biomedical research, including ethical concerns, high costs, and poor translational relevance to human biology, have driven increasing interest in computational simulation models. These models encompass in silico approaches, pharmacokinetic/pharmacodynamic frameworks, molecular simulations, and organ-on-chip technologies, offering greater precision in replicating human physiological and pathological processes.

**Methods:**

A systematic review was conducted to examine the role of computational simulation models as alternatives to traditional animal-based research. Relevant literature on their applications, predictive accuracy, translational value, and alignment with ethical research practices was analyzed.

**Results:**

Computational models were found to bridge critical gaps in predictive accuracy and translational relevance, supporting drug development pipelines, reducing late-stage failures, and enhancing opportunities for personalized medicine. Additionally, their capacity to reduce reliance on animal models aligns with global ethical initiatives promoting humane and sustainable research practices.

**Discussion:**

Simulation-based approaches represent a transformative opportunity for biomedical research. While their potential to reshape drug development and improve health outcomes is evident, challenges such as standardization, scalability, and regulatory integration remain. Addressing these barriers will be essential to fully realize the potential of computational simulation models in replacing or reducing animal testing and advancing human-centered biomedical innovation.

**Systematic Review Registration:**

identifier, INPLASY2024110028.

## Introduction

**FIGURE 1:**
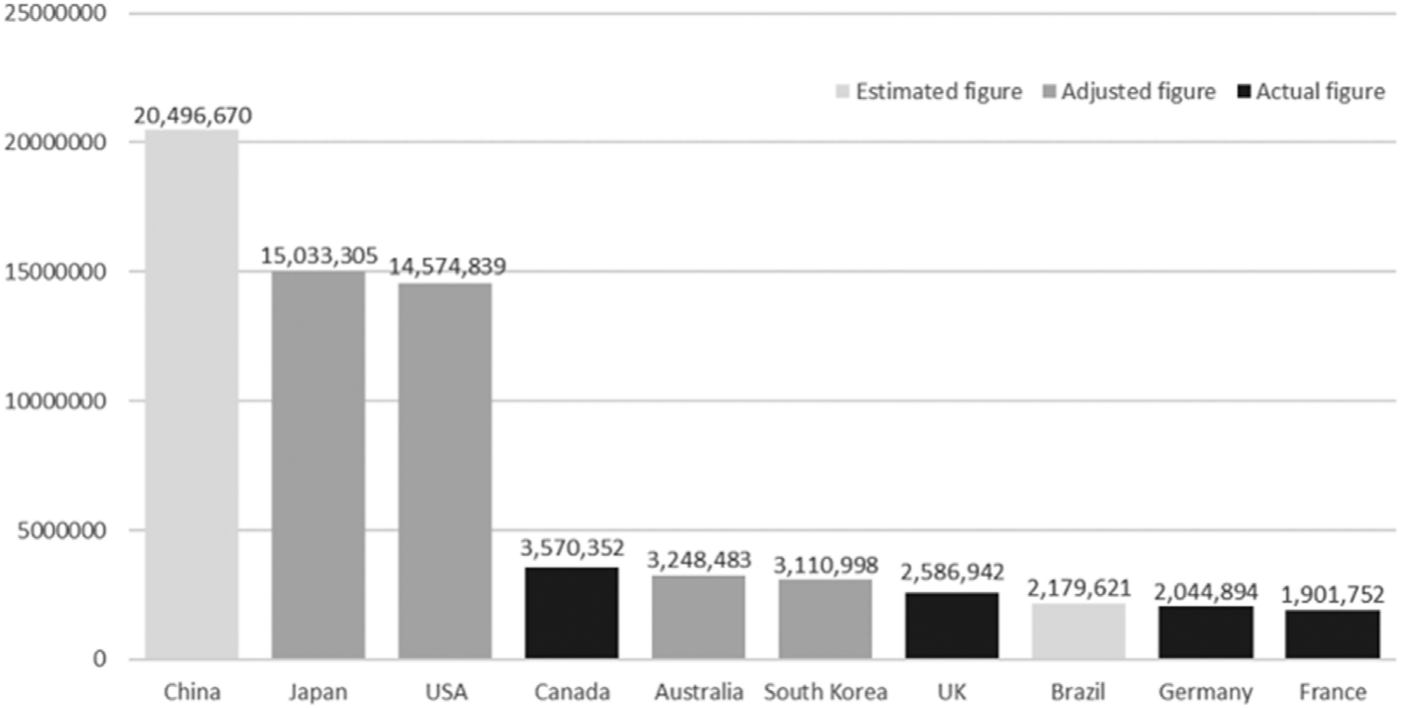
The top ten animal-using countries based on actual, adjusted and estimated figures. The figures represent the numbers of procedures with animals. Figures were adjusted according to the European Union (EU) definitions and estimated figures were derived from the statistical model. Taken from Taylor and Alvarez (2019) under the Creative Commons Attribution-NonCommercial 4.0 License.

The use of animal models has long been integral to drug testing and biomedical research, providing a foundation for understanding disease mechanisms, evaluating therapeutic efficacy, and ensuring safety before clinical trial (Kinter et al., 2021; Domínguez-Oliva et al., 2023; Franco, 2013; Fernandes et al., 2025). These models have enabled researchers to explore complex biological systems, predict pharmacological responses, and assess potential adverse effects (Chang and Grieder, 2024; Soufizadeh et al., 2024). However, animal models are increasingly recognized as having substantial limitations that hinder their broader utility in modern biomedical research. One of the most pressing issues is the ethical concern surrounding the use of animals in experimental research (Kiani et al., 2022; Andersen and Winter, 2019; Kekeçoğlu and Kocaman Kalkan, 2024). The welfare of animals used in studies has become a significant point of contention, prompting calls for more humane and ethically justifiable research practices (Neziri et al., 2024; Grandin and Deesing, 2022; Browning, 2022; Richter, 2024; McGill and Threadgill, 2023; Kaplan et al., 2024). In addition to ethical considerations, animal research demands substantial resources, including specialized facilities and skilled personnel, which can constrain the scale and duration of studies (Mertz et al., 2024; Fontana et al., 2021; Neves et al., 2024; Saeidnia et al., 2015; Khabib et al., 2022; Schluter, 2024; Rudroff, 2024). The scale of animal use remains high globally, as reflected in reporting frameworks aligned with international standards (Figure 1) (Taylor and Alvarez, 2019). A major limitation lies in the poor translational applicability of animal data to human biology (Rhrissorrakrai et al., 2015). Interspecies differences in genetics, immune function, and metabolism often lead to discrepancies that undermine the predictive value of preclinical findings (Singh et al., 2024; Mekada and Yoshiki, 2021; Fontaine and Davis, 2016; Pizzollo et al., 2018; Atkins et al., 2020). As a result, many drugs that perform well in animal studies ultimately fail in human trials, contributing to high attrition rates and increased development costs (Van Norman, 2020; Van Norman, 2019).

Technological advancements have opened new avenues for innovative alternatives, with simulation models emerging as a transformative approach in biomedical research (Nithin et al., 2023; Ingber, 2020; Madden et al., 2020; Huang et al., 2021; Craig et al., 2023). These models encompass a broad spectrum of technologies, including *in silico* computational methods, and organ-on-chip systems (Agamah et al., 2020; Stavrou et al., 2023; Kutluk et al., 2023; Alonso-Roman et al., 2024; Koyilot et al., 2022; Abadi et al., 2020) (Table 1). Designed to replicate human physiological and pathological processes with remarkable precision, simulation models offer the potential to overcome many of the challenges associated with traditional animal models (Madden et al., 2020). In silico methods utilize sophisticated computational algorithms to simulate complex biological systems, enabling predictions of drug behavior, efficacy, and safety across multiple scenarios (Hasan et al., 2023; Saldanha et al., 2023). These models integrate data from various sources, including genomics, proteomics, and pharmacokinetics, to construct virtual representations of human biology (Shaker et al., 2021). Meanwhile, organ-on-chip platforms have revolutionized experimental research by recreating the structural and functional microenvironment of human tissues (Srivastava et al., 2024; Ma et al., 2021). These microscale systems combine bioengineering and cellular biology to replicate key aspects of organ physiology, allowing for dynamic and high-fidelity studies of drug responses, toxicity, and disease mechanisms (Cho et al., 2023; Huang et al., 2024; Monteduro et al., 2023).

**TABLE 1:** Comparison of key simulation models in drug testing and biomedical research.

Model type	Validation metrics	Endpoints	Strengths	Weaknesses	Key references
Pharmacokinetics/Pharmacodynamics (PK/PD)	R^2^, MAE, Clinical Concordance	Drug absorption, distribution, metabolism, elimination	Mechanistic clarity, human-relevant, scalable	Requires extensive data, may lack immunological complexity	Chan et al. (2019), Van Tongeren et al. (2021), Kammala et al. (2023)
In Silico Molecular Docking	Binding Affinity (kcal/mol), AUC	Drug-target binding, toxicity prediction	High-throughput, cost-effective, guides compound prioritization	Dependent on structural data, limited by docking assumptions	Adnan et al. (2020), Alam et al. (2021), Alauddin et al. (2024), Paul et al. (2021), Rani et al. (2024)
Machine Learning (ML) & Deep Learning (DL)	Accuracy, AUC, MCC, F1-score	Toxicity classification, drug absorption, structure prediction	Data-driven, high accuracy, interpretable (with explainability)	Requires quality data, risk of overfitting, black-box nature	Di Stefano et al. (2024), Feng et al. (2021), Sharma et al. (2023), Chen et al. (2023), Li et al. (2023), Zhou et al. (2024), Im et al. (2023)
Organ-on-Chip	TEER Values, Permeability Correlation (r)	Tissue permeability, drug response, barrier function	Mimics physiological microenvironments, real-time response	Expensive setup, limited scalability, technical complexity	Kim et al. (2023), Kammala et al. (2023)
Physiologically-Based Pharmacokinetic Models (PBPK)	Fold-Deviation from Clinical PK, R^2^	Plasma concentration, clearance prediction	Accurate exposure simulation, reduces *in vivo* studies	Needs *in vitro* data integration, species-specific limitations	Chan et al. (2019), Van Tongeren et al. (2021), Leedale et al. (2018)
Generative Models (such as AnimalGAN, and TransOrGAN)	Similarity Scores, Consistency with Real Data	Clinical pathology, transcriptomic variability	Handles biological variability, predicts unseen outcomes	Complex training, potential overfit, validation still evolving	Chen et al. (2023), Li et al. (2023)

Beyond ethical advantages, simulation models enhance predictive accuracy and narrow the translational gap common with animal studies (Geng et al., 2024; Laman et al., 2017). As a result, simulation models enable researchers to predict drug behavior, efficacy, and safety more effectively, minimizing the risk of late-stage failures in clinical trials (Alqahtani, 2023; Alves et al., 2021). Additionally, by providing a scalable and resource-efficient alternative, these models can lower the costs associated with preclinical research and drug testing (Shaker et al., 2021; Franzen et al., 2019; Arsène et al., 2023). The integration of human-relevant data generated by simulation models has profound implications for the efficiency of drug development pipelines (Agamah et al., 2020; Si et al., 2021; Azizgolshani et al., 2021; Na et al., 2020; Salahshoori et al., 2024). Moreover, simulation models allow researchers to test multiple variables simultaneously, accelerating the discovery and optimization of novel therapies while maintaining high levels of reproducibility and control (Cronin et al., 2023; Riggs et al., 2021; Solim et al., 2022).

Additionally, simulation models align with global initiatives advocating for the refinement, reduction, and replacement of animal use in research, as supported by regulatory bodies, funding agencies, and international ethical guidelines (National Institute of Environmental Health Sciences, 2025; Singer and Akhtar, 2024; Hutchinson et al., 2022). The U.S. Food and Drug Administration (FDA) has been at the forefront of these efforts, actively promoting the integration of simulation technologies into drug development and regulatory processes. For instance, the FDA’s Predictive Toxicology Roadmap emphasizes the use of computational models, *in silico* simulations, and other non-animal methodologies to improve the prediction of drug safety and efficacy (FDA, 2017). The agency has also launched initiatives such as the Innovative Science and Technology Approaches for New Drugs (ISTAND) program, which supports the qualification of novel tools, including simulation models, to complement traditional approaches (FDA, 2024). Moreover, the FDA Modernization Act 2.0 has recently expanded the regulatory framework to include alternative methods, such as organ-on-chip systems and computational modeling, as acceptable tools for drug testing (Han, 2023; Adashi et al., 2023). These initiatives demonstrate the FDA’s commitment to advancing simulation models as part of a broader effort to enhance the scientific and ethical standards of biomedical research.

However, the integration of simulation models into mainstream research and regulatory frameworks remains limited. Critical challenges such as validating and standardizing these models, ensuring scalability, and establishing reliability in capturing complex biological systems must be addressed (Avramouli, 2020) (Table 1). Adopting simulation models also necessitates a paradigm shift in research, fostering multidisciplinary collaboration among bioengineers, computational scientists, and regulatory experts.

The objective of this systematic review is to provide a comprehensive evaluation of the current landscape of simulation models in drug testing and biomedical research. It examines their applications, strengths, and limitations while exploring their future potential as viable alternatives to animal models. By synthesizing evidence from diverse sources, the review highlights the transformative role simulation models can play in advancing biomedical science while addressing the key challenges that must be overcome for broader adoption.

## Methods

This systematic review aimed to comprehensively evaluate the potential of simulation models as alternatives to animal models in drug testing and biomedical research. The study followed a predefined protocol registered on the International Platform of Registered Systematic Review and Meta-Analysis Protocols (INPLASY) under registration number INPLASY2024110028, ensuring methodological rigor and transparency throughout the review process.

### Search strategy

A systematic search was conducted across databases PubMed (MEDLINE), Embase, Scopus, and ScienceDirect. Keywords and Medical Subject Heading (MeSH) terms such as “simulation models,” “*in silico* models,” “computational simulations,” “animal alternatives,” “drug testing,” and “biomedical research” were employed in various combinations using Boolean operators. As narrative or systematic review articles were available covering studies up to 2017, the searches were performed between 1 January 2018 to 1 September 2024.

The selection of search terms was guided by an iterative strategy that integrated subject matter expertise, preliminary scoping searches, and structured vocabularies, including MeSH (Medical Subject Headings) for PubMed and Emtree for Embase. Core concepts central to the study such as “*in silico* models,” “drug toxicity,” and “organ on chip” were initially identified and systematically expanded using relevant synonyms, hierarchical terms, and Boolean logic to optimize search precision and recall. Keyword refinement was informed by term frequency analysis and relevance assessments conducted during pilot searches, ensuring alignment with current terminologies and indexing practices across databases.

### Eligibility criteria

Studies were included in this review if they focused on computational simulations, *in silico* models, or predictive algorithms designed to assess drug responses, toxicity, or disease progression. Eligible models included pharmacokinetics/pharmacodynamics (PK/PD) models, systems biology frameworks, molecular simulations, virtual organs, tissue models, and multi-scale simulations. Applications of these models in drug testing (such as efficacy, safety, toxicity), disease modeling (such as cancer, diabetes, neurodegenerative diseases), and other biomedical research areas (such as organ function simulations, virtual clinical trials) were considered relevant. Additionally, studies were required to provide direct comparisons between simulation models and traditional animal models, focusing on predictive accuracy, reliability, and ethical or cost considerations.

Exclusion criteria encompassed studies solely focused on traditional animal models or *in vitro* approaches without any simulation component. Reviews, commentaries, and opinion articles lacking primary data were excluded, as were studies with insufficient methodological detail to allow thorough assessment. These criteria ensured a focused and meaningful synthesis of the available evidence.

### Study selection

The study selection procedure was performed in three sequential stages: title screening, abstract screening, and full-text assessment. Three independent reviewers (AH, HA, and MS) systematically evaluated all retrieved records to determine their eligibility according to the inclusion criteria. Inter-rater reliability was assessed at each stage using Cohen’s kappa (κ) statistic. Interpretation of κ values followed established thresholds: values < 0 indicated no agreement; 0.00–0.20 denoted slight agreement; 0.21–0.40, fair agreement; 0.41–0.60, moderate agreement; 0.61–0.80, substantial agreement; and 0.81–1.00, almost perfect agreement. Consistent with prior literature, a κ value exceeding 0.60 was considered indicative of substantial agreement, thereby supporting the methodological rigor of the selection process. Any discrepancies between reviewers were resolved through discussion with another reviewer or senior authors (RM and KH).

### Data extraction

Data from the included studies were systematically extracted using a structured template to ensure consistency and comprehensiveness. For each study, detailed characteristics of the simulation models were captured, such as model type, computational methods, algorithms, input parameters, and validation strategies. The model applications were categorized according to specific disease areas such as cardiovascular, neurological, metabolic, and infectious disorders allowing for domain-relevant insights into drug testing, safety profiling, and disease modeling. Additionally, outcomes were analyzed for predictive accuracy, translational relevance, cost-effectiveness, reduction in animal usage, and alignment with ethical considerations. This rigorous extraction process ensured a thorough evaluation of the studies’ contributions to the field.

### Risk of bias and quality assessment

The methodological quality and potential for bias in the included studies were appraised using standardized instruments developed by the Joanna Briggs Institute (JBI). The choice of tool was based on study design and adapted as appropriate for computational research contexts. For quasi-experimental studies, the JBI Critical Appraisal Checklist for Quasi-Experimental Studies was utilized. This tool systematically evaluates internal validity across multiple domains, including the clarity of cause-effect relationships, presence and comparability of control groups, consistency in intervention delivery, reliability of outcome measurement, and adequacy of follow-up.

For studies evaluating diagnostic test accuracy using computational models, a modified version of the JBI Critical Appraisal Checklist for Diagnostic Test Accuracy Studies was utilized. This adaptation addressed core domains relevant to computational methodologies, such as dataset representativeness, class balancing, exclusion criteria, independence of test generation, threshold pre-specification, reference standard validity, blinding of training and evaluation, dataset partitioning, validation consistency, and data completeness.

Each study was independently reviewed by three evaluators (AH, HA, and MS), with the findings compiled into a comprehensive risk of bias table. Any discrepancies between reviewers were resolved through discussion and consensus or discussion with the senior authors (RM and KH), ensuring a robust and unbiased assessment process.

### Data synthesis

Given the anticipated heterogeneity in study designs and outcomes, a narrative synthesis was conducted to provide a structured and comprehensive analysis. Extracted data were categorized by application type, such as drug testing and disease modeling, and systematically analyzed to identify patterns, strengths, and limitations of simulation models in comparison to traditional animal models. Key outcomes, including predictive accuracy, translational relevance, cost-effectiveness, and ethical considerations, were emphasized. Comparative analyses further delineated areas where simulation models demonstrate significant advantages and highlighted opportunities for improvement and further development.

## Results

**FIGURE 2:**
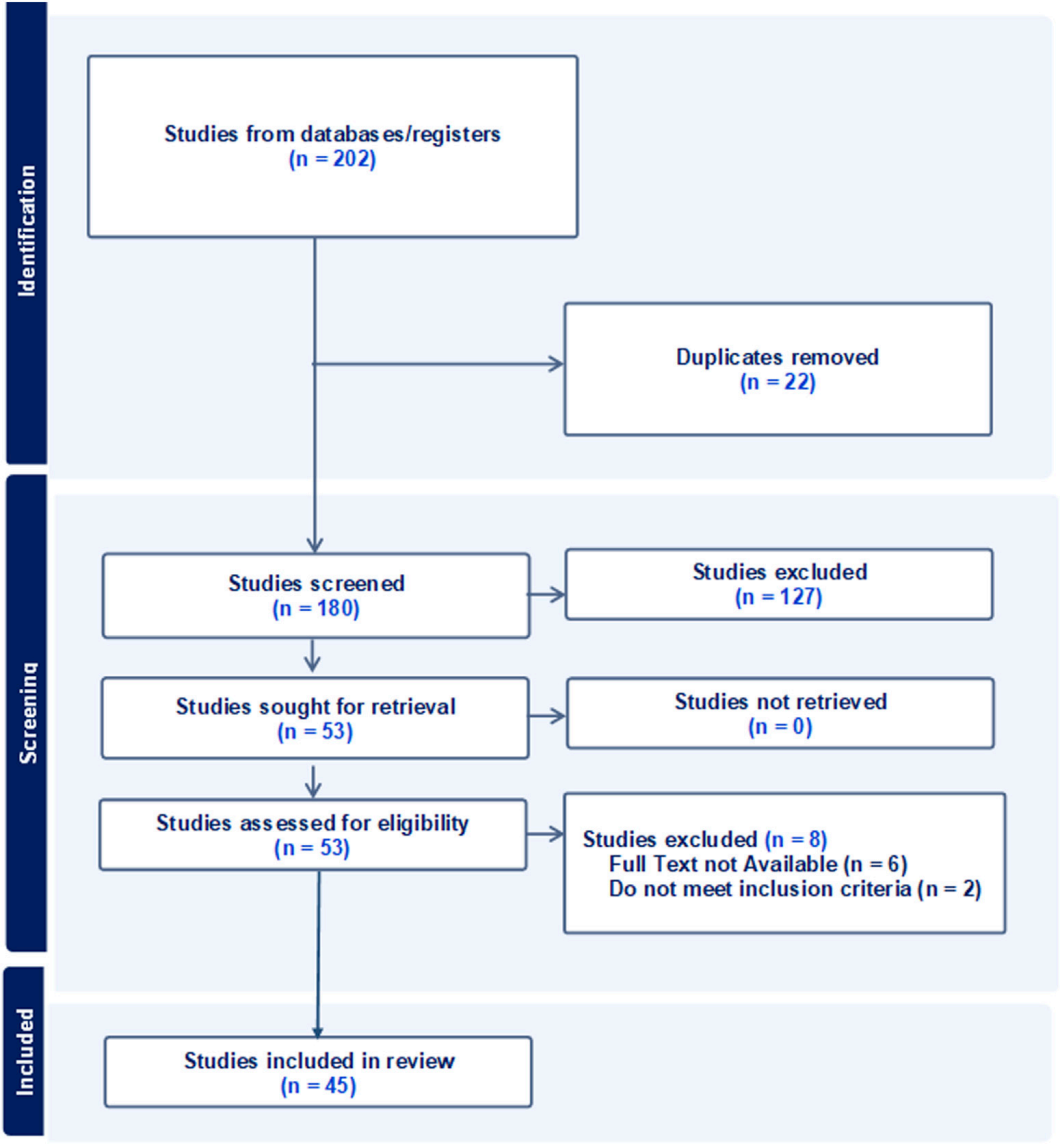
A PRISMA (Preferred Reporting Items for Systematic Reviews and Meta-Analyses) flow diagram of study selection process.

A total of 202 records were identified through database searches. After removing 22 duplicates, 180 records were screened for relevance. Of these, 127 studies were excluded based on titles and abstracts. The remaining 53 studies were assessed for eligibility. Eight full-text articles were excluded, six due to unavailability and two for not meeting the inclusion criteria. Ultimately, 45 studies were included in the qualitative synthesis. The study selection process is detailed in the PRISMA flow diagram (Figure 2). A summary of the studies’ characteristics is shown in Supplementary Table S1.

Inter-rater reliability was evaluated using Cohen’s kappa (κ) statistics to quantify the level of agreement between reviewers at each stage of study selection. The analysis demonstrated substantial agreement, with a κ value of 0.746 for title and abstract screening and 0.866 for full-text screening, indicating a high degree of consistency and methodological rigor in the screening process.

### Risk of bias assessment

**FIGURE 3:**
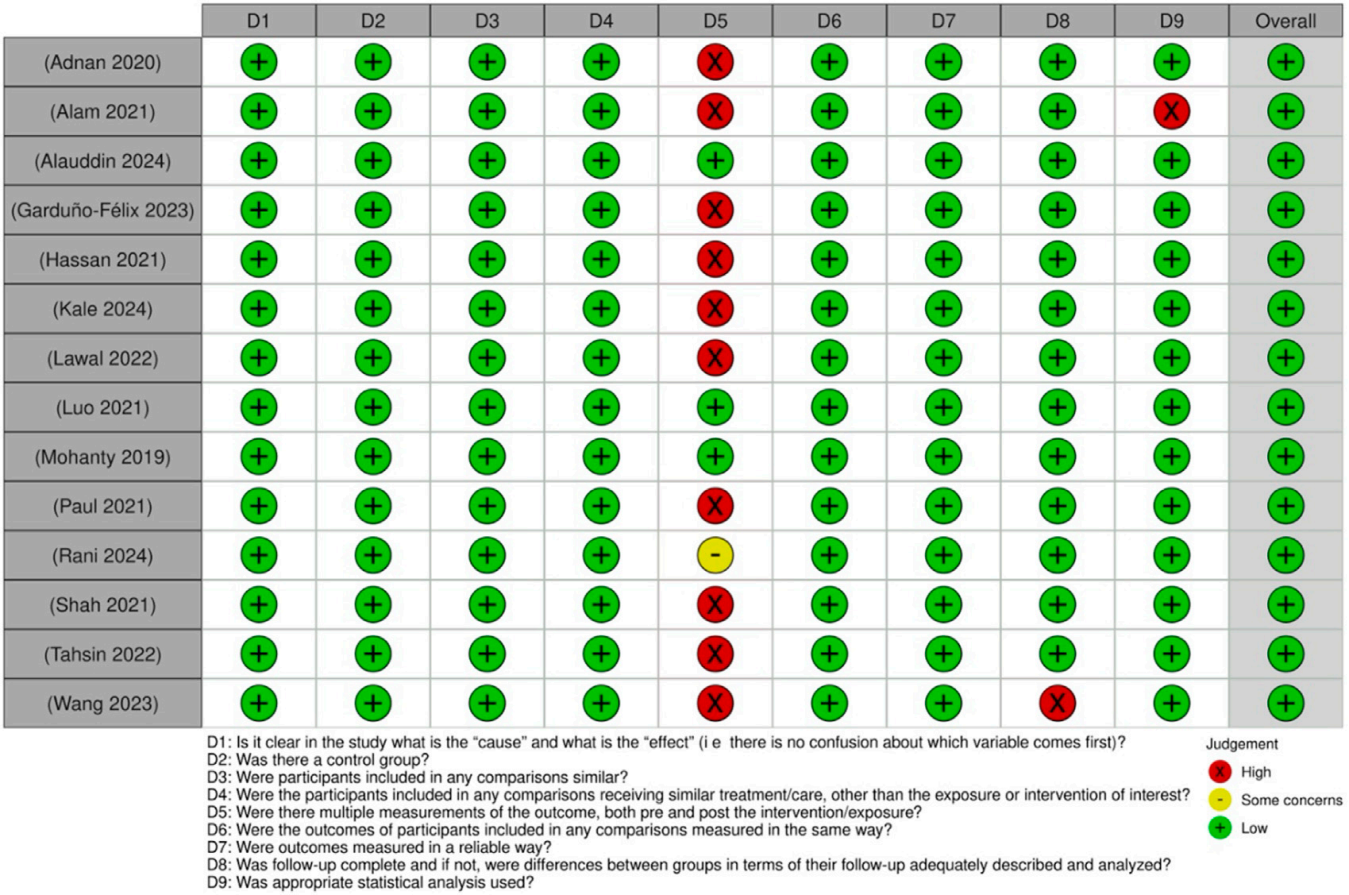
Risk of bias assessment of the included quasi-experimental studies: This figure presents the risk of bias assessment of the included studies based on the Joanna Briggs Institute (JBI) Critical Appraisal Checklist for Quasi-Experimental Studies. The color coding represents the level of bias: green denotes low risk of bias, yellow indicates unclear risk, and red suggests high risk of bias.

The risk of bias across the included studies was systematically evaluated using appropriate tools tailored to each study design. For quasi-experimental studies, domains assessed included selection bias, performance bias, detection bias, attrition bias, and reporting bias (Figure 3). The majority of these studies demonstrated a low to moderate risk of bias, with some concerns arising mainly in blinding and confounding domains.

**FIGURE 4:**
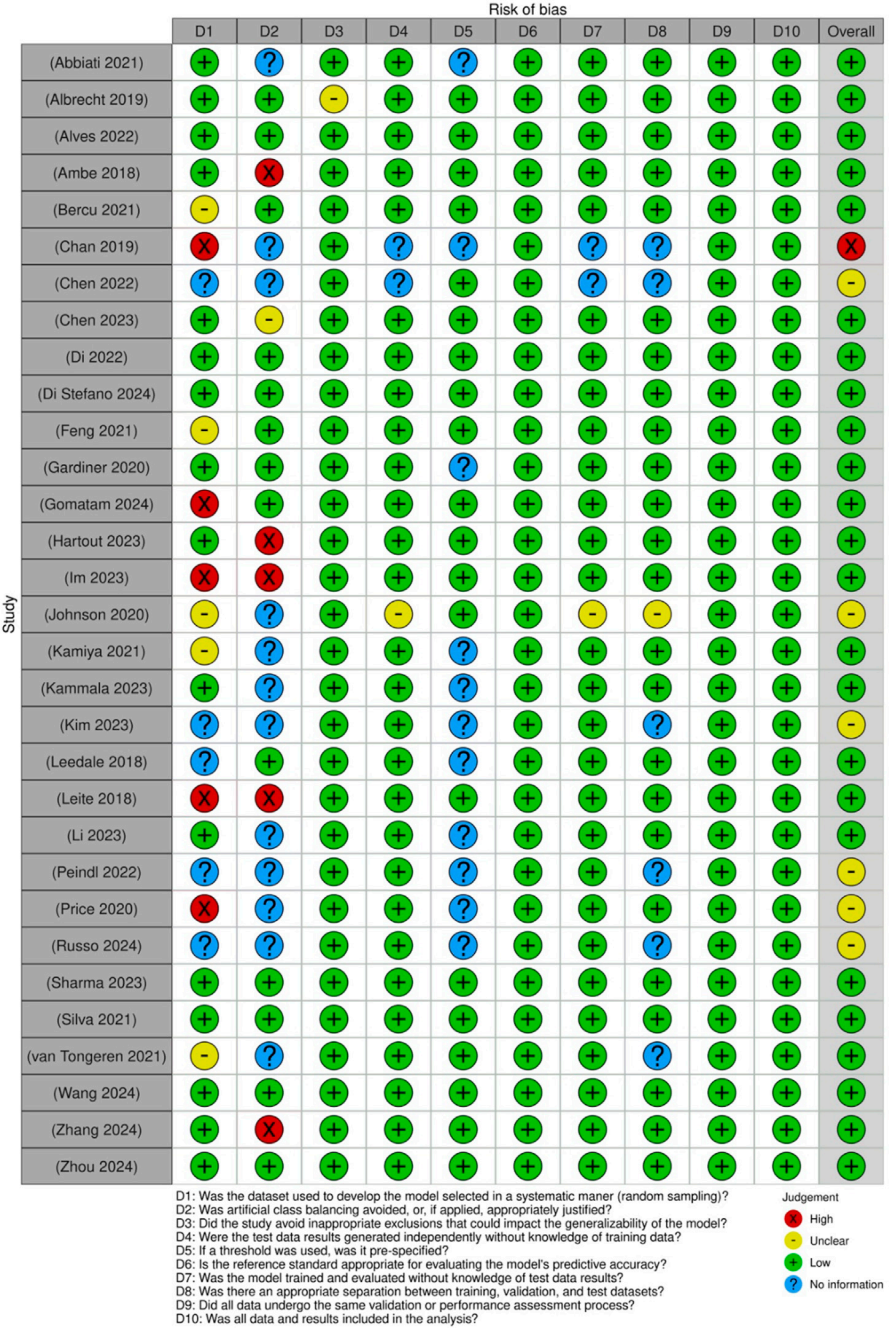
Risk of bias assessment of the included diagnostic test accuracy studies: This figure presents the risk of bias assessment of the included studies based on the Joanna Briggs Institute (JBI) Critical Appraisal Checklist for Diagnostic Test Accuracy Studies. The color coding represents the level of bias: green denotes low risk of bias, yellow indicates unclear risk, red suggests high risk of bias, and blue represents information not available.

For studies assessing diagnostic test accuracy, a modified tool was used to accommodate computational approaches (Figure 4). Key areas of concern included patient selection, index test conduct, reference standard applicability, and flow and timing. While most studies scored favorably, some exhibited potential bias related to the lack of clarity in test procedures and standardization.

## Applications of computer modelling for various disorders

### Gastrointestinal and hepatic disorders

In the field of gastrointestinal and hepatic disorders, recent research has increasingly emphasized the integration of computational simulations with *in vivo* experimentation to enhance both the discovery and mechanistic validation of therapeutic compounds, particularly those derived from natural sources. This dual-modality approach allows researchers not only to observe pharmacodynamic outcomes in animal models but also to interrogate molecular interactions at the target-receptor level using *in silico* techniques. A study evaluated the methanolic leaf extract of Cuscuta reflexa in murine models of diarrhea and oxidative stress (Adnan et al., 2020). The extract produced a statistically significant reduction in castor oil-induced diarrhea, while biochemical assays revealed decreased malondialdehyde (MDA) levels and increased catalase (CAT) and superoxide dismutase (SOD) activity, indicating a strong antioxidant response. Subsequent molecular docking analysis revealed that active constituents such as kaempferol and quercetin exhibited high binding affinities to muscarinic acetylcholine receptors and cyclooxygenase-2 (COX-2), with docking scores ranging from −8.1 to −9.4 kcal/mol, supporting their roles in modulating intestinal motility and inflammatory pathways (Adnan et al., 2020).

Similarly, the antidiarrheal, antimicrobial, and antioxidant potential of Colocasia gigantea leaf extract was demonstrated in a separate study (Alam et al., 2021). *In vivo* assays in mice showed dose-dependent reductions in diarrhea frequency and improvements in oxidative stress markers. In silico molecular docking further substantiated these findings by demonstrating that bioactive phytochemicals such as β-sitosterol and orientin showed strong binding to targets including toll-like receptor 4 (TLR4) and inducible nitric oxide synthase (iNOS), with predicted binding energies exceeding −9 kcal/mol. These computational insights not only corroborated the *in vivo* effects but also elucidated probable molecular mechanisms, thereby accelerating the transition from empirical pharmacological observations to target-specific drug development strategies.

Beyond the discovery of therapeutic mechanisms, the application of computational modeling in gastrointestinal and hepatic research has increasingly shifted toward predictive toxicology and pharmacokinetics. This paradigm is exemplified by a study that utilized a support vector machine (SVM)-based classifier trained on pharmacokinetic and toxicological datasets to predict drug-induced liver injury (DILI) across a large panel of compounds (Albrecht et al., 2019). The model achieved a balanced accuracy of 81 percent and demonstrated the ability to classify DILI risk categories in concordance with human clinical data. In a complementary study, an ensemble machine learning (ML) approach was applied by incorporating random forest (RF) and deep learning (DL) models to predict hepatocellular hypertrophy induced by chemical exposure (Ambe et al., 2018). By using over 400 molecular descriptors and toxicogenomic data, the models achieved sensitivity values exceeding 85 percent, indicating strong predictive capability.

In the domain of intestinal pharmacokinetics, a study developed a hybrid in vitro–in silico framework by integrating pH-dependent Caco-2 permeability assays with Light Gradient Boosting Machine (LightGBM) models to predict intestinal absorption of 219 structurally diverse compounds (Kamiya et al., 2021). Their model achieved a coefficient of determination (R^2^) of 0.81 and a mean absolute error (MAE) of 0.37 log units when predicting human jejunal effective permeability (Peff), outperforming traditional regression-based approaches. Importantly, the inclusion of pH variability in both experimental and computational models allowed for improved simulation of the gastrointestinal environment, enhancing translational relevance.

### Cardiovascular diseases

Cardiovascular diseases (CVDs) remain the leading cause of morbidity and mortality worldwide, necessitating the continuous development of effective, safe, and accessible therapeutic interventions. Traditional drug discovery approaches in this domain have been time-consuming, resource-intensive, and heavily reliant on animal experimentation. However, the integration of computational modeling into cardiovascular research has revolutionized early-phase therapeutic screening and mechanistic exploration. In silico approaches, particularly molecular docking and simulation techniques, enable rapid identification of promising drug candidates by modeling interactions between bioactive compounds and disease-relevant targets, significantly reducing the experimental burden. A study highlighted the critical role of computer modeling in drug discovery by demonstrating how molecular docking simulations can effectively guide and streamline the identification of therapeutic candidates (Alauddin et al., 2024). In this study, over 50 peptides derived from enzymatically hydrolyzed soymilk proteins were screened *in silico* against angiotensin-converting enzyme (ACE), a central regulator of blood pressure. The tetrapeptide FFYY (Phe-Phe-Tyr-Tyr) showed the strongest inhibitory potential, with a docking score of −10.2 kcal/mol, indicating a high binding affinity. Structural analysis revealed that FFYY formed key interactions with ACE residues His383, Glu384, and Tyr523, suggesting a competitive inhibition mechanism. This computational prediction informed the selection of FFYY for *in vivo* testing, where oral administration at 10 mg/kg/day over a 28-day period led to a 21 mm Hg reduction in systolic blood pressure (SBP) in spontaneously hypertensive rats. For comparison, a reference group treated with captopril, a clinically approved first-generation ACE inhibitor known for its potent vasodilatory and antihypertensive effects, exhibited a 23 mm Hg reduction in SBP, demonstrating the comparable efficacy of FFYY.

In addition to lowering blood pressure, the FFYY-treated group exhibited a 35 percent reduction in plasma angiotensin II levels, a 30 percent decrease in serum aldosterone, and enhanced oxidative stress profiles, including a 28 percent increase in superoxide dismutase (SOD) activity and a 25 percent decrease in malondialdehyde (MDA) levels. These biochemical changes suggest that FFYY exerts a multifaceted cardioprotective effect, not only through Renin–Angiotensin–Aldosterone System (RAAS) modulation but also by mitigating oxidative stress, which is known to contribute to vascular damage and hypertension progression.

Histological examination of cardiac and renal tissues provided further evidence of therapeutic benefit and safety. Unlike untreated hypertensive controls, which showed clear signs of vascular congestion, myocardial hypertrophy, and glomerular distortion, the FFYY-treated rats maintained normal tissue architecture with no evidence of fibrosis, cellular necrosis, or inflammatory infiltration. This indicates that FFYY not only reduces hemodynamic stress but may also prevent structural damage to target organs commonly affected by chronic hypertension. By prioritizing bioactive compounds through *in silico* modeling, this study minimized the need for broad-spectrum animal testing while offering a mechanistically informed, ethically advantageous, and cost-effective pipeline for antihypertensive drug discovery.

### Diabetes and related disorders

In the category of diabetes and related metabolic disorders, recent research has increasingly relied on computational tools, particularly molecular docking, to complement *in vivo* and *in vitro* methods in the evaluation of novel therapeutic candidates. Across multiple studies, plant-derived compounds have emerged as particularly promising candidates for diabetes management, often demonstrating hypoglycemic, insulin-sensitizing, and antioxidant effects in animal models. For example, a comprehensive study explored extracts from small cardamom and yellow mustard seeds (Paul et al., 2021). These extracts were found to significantly reduce fasting blood glucose levels in streptozotocin-induced diabetic rats. In silico docking analyses further revealed that key bioactive components such as 1,8-cineole and allyl isothiocyanate exhibited strong binding affinities to targets like alpha-amylase and alpha-glucosidase, with binding energies of −8.5 kcal/mol and −9.1 kcal/mol, respectively. These enzymes are involved in the breakdown of complex carbohydrates into glucose, and their inhibition is a well-validated therapeutic strategy to reduce postprandial blood glucose spikes.

Similarly, the antidiabetic potential of Azanza garckeana was investigated in streptozotocin-induced glycemic-impaired rats (Lawal et al., 2022). The administration of the plant extract led to a marked improvement in glycemic control, insulin sensitivity, and pancreatic histoarchitecture. Docking studies revealed high binding affinities of the major phytoconstituents to dipeptidyl peptidase-IV (DPP-IV), an enzyme responsible for the degradation of incretin hormones such as GLP-1, which play a critical role in insulin secretion. The inhibition of DPP-IV by these compounds was consistent with observed *in vivo* enhancements in circulating insulin levels and beta-cell regeneration.

The pharmacological effects of Gynura procumbens, a plant traditionally used in Southeast Asian medicine, were explored in a different study (Tahsin et al., 2022). That study employed a combination of animal models and molecular docking to evaluate antidiabetic efficacy. Treatment with Gynura extracts resulted in significant reductions in fasting blood glucose, total cholesterol, and triglycerides. Docking simulations showed strong interactions between the plant’s flavonoids and several key metabolic proteins, including glucokinase and peroxisome proliferator-activated receptor gamma (PPARγ), that are involved in glucose utilization and lipid metabolism. Binding energy values for these interactions were reported between −7.2 and −9.8 kcal/mol, indicating high affinity and potential for pharmacological action.

Another study examined the cardioprotective and antidiabetic properties of Terminalia arjuna in diabetic rats (Mohanty et al., 2019). Their investigation combined *in vitro* enzyme assays, *in vivo* efficacy tests, and molecular docking of its triterpenoids and flavonoids. The study identified potent inhibition of DPP-IV, with one compound, arjunolic acid, displaying a docking score of −10.4 kcal/mol. This interaction correlated with observed improvements in cardiac function and glycemic control in the experimental animals, highlighting the systemic benefits of the compound beyond glucose regulation alone.

In contrast to the traditional ligand–enzyme docking approaches, a fundamentally different paradigm was introduced by applying protein language modeling to the field of computational immunology, specifically focusing on MHC class II peptide presentation, a critical aspect of immune recognition and autoimmunity in diabetes (Hartout et al., 2023). Their model, AEGIS, utilizes a transformer-based DL architecture trained on large-scale mass spectrometry-derived immunopeptidomic datasets, enabling the identification of peptide–MHC binding preferences with unprecedented accuracy. The model achieved an area under the curve (AUC) greater than 0.95 for human HLA class II alleles and 0.88 for non-obese diabetic (NOD) mice, significantly outperforming established models such as NetMHCIIpan and MARIA. Importantly, AEGIS demonstrated high predictive fidelity for disease-relevant epitopes, including those derived from insulin and glutamic acid decarboxylase, which are central to the pathogenesis of type 1 diabetes. Unlike conventional docking, which is limited by structural data availability and conformational sampling constraints, AEGIS captures long-range sequence dependencies and contextual motifs using self-attention mechanisms; this allows it to generalize across alleles and species. This approach enables rapid and scalable prioritization of candidate autoantigens and supports the rational design of tolerogenic peptide therapies, reducing reliance on *in vitro* binding assays and preclinical animal models. By exemplifying the shift toward high-dimensional, data-driven modeling in immunology, AEGIS highlights the transformative potential of artificial intelligence to accelerate therapeutic discovery in autoimmune diabetes while enhancing biological relevance and ethical viability.

### General toxicity and pharmacokinetic prediction

The field of toxicology and pharmacokinetic prediction has undergone transformative growth with the increasing integration of artificial intelligence (AI) and computational modeling, driven by the exponential availability of high-quality chemical, biological, and toxicological datasets. These advancements have enabled the development of sophisticated *in silico* platforms capable of predicting complex toxicity endpoints and drug disposition characteristics with unprecedented accuracy and efficiency. Central to this progress is the shift toward ML- and DL-based models, which are now routinely used to predict toxicological outcomes directly from molecular structure, offering cost-effective, scalable, and ethically favorable alternatives to traditional animal testing.

**FIGURE 5:**
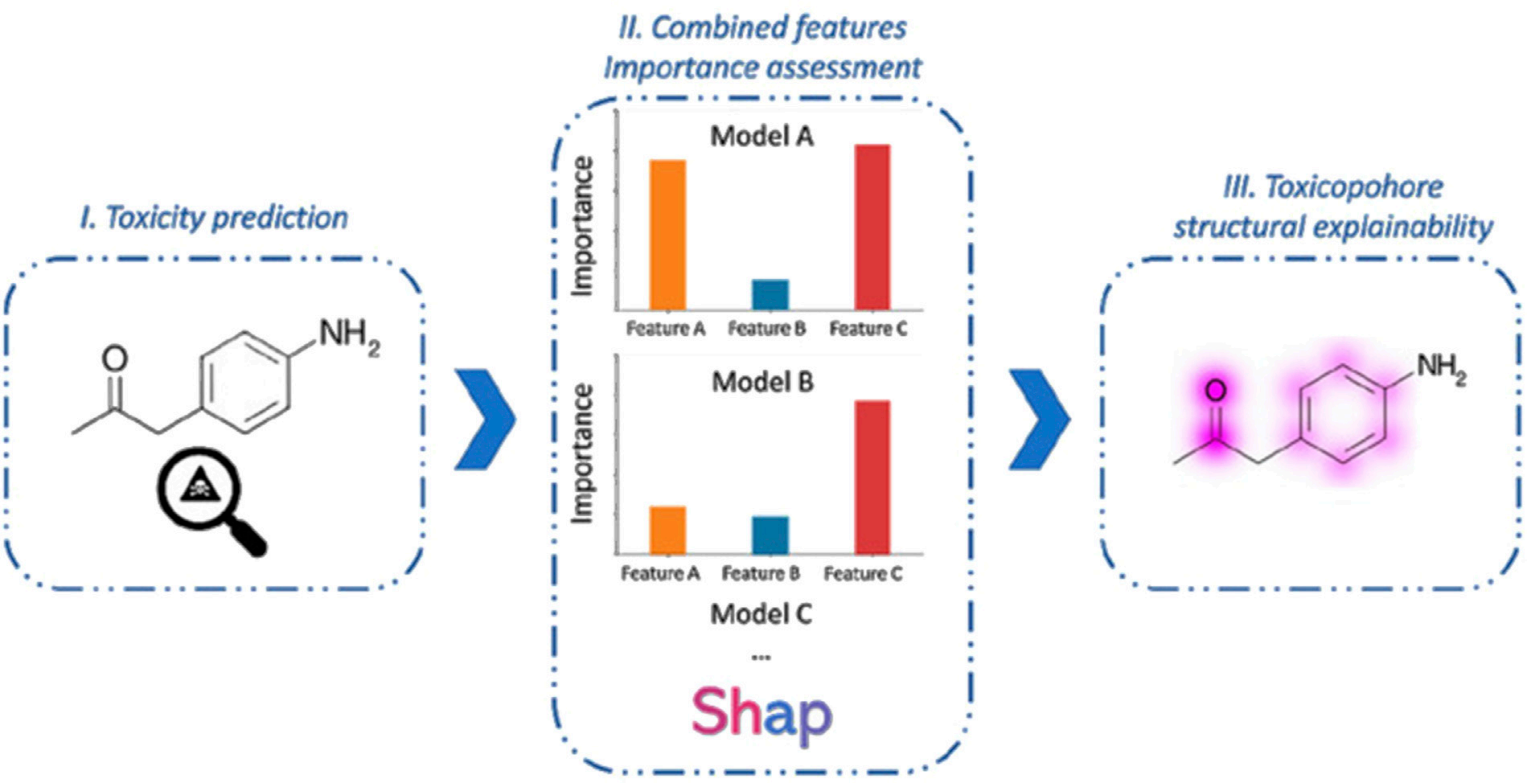
SHAP method workflow: (I) Toxicity prediction; (II) Assessing features importance obtained from the models of the best consensus combination; (III) Retro-mapping of feature impact to highlight the moieties that strongly influence the toxicity prediction. Taken from Di Stefano et al. (2024) under the Creative Commons License (CC-BY 4.0).

A prominent example of this paradigm is VenomPred 2.0, a multi-endpoint toxicity prediction platform that applies ensemble machine learning algorithms integrated with explainable AI methods such as SHAP (SHapley Additive exPlanations) (Figure 5) (Di Stefano et al., 2024). This model has successfully identified structural features associated with adverse effects, including hepatotoxicity and androgenicity, achieving high predictive performance with a Matthews correlation coefficient (MCC) of up to 0.94. Similarly, an ensemble ML model was developed that used molecular fingerprints to predict reproductive toxicity, achieving an AUC of 0.920 (Feng et al., 2021). That model provided transparent feature importance rankings, enabling mechanistic interpretations of toxicity pathways and enhancing model trustworthiness. Building on these advancements, a multi-task deep neural network has been designed to predict a wide range of clinical toxicities across pharmacological classes (Sharma et al., 2023). This model not only achieved an exceptional AUC-ROC of 0.99 but also incorporated contrastive explanation methods to identify substructural features contributing to toxicity, pushing the boundaries of interpretability in deep learning applications.

Beyond standalone predictive performance, there is a growing emphasis on benchmarking and validating these models in regulatory contexts. A study evaluated the fitness-for-purpose of acute oral toxicity QSAR models and demonstrated that such models correctly or conservatively predicted Globally Harmonized System (GHS) toxicity categories for approximately 95 percent of tested compounds (Bercu et al., 2021). Balanced accuracy across all hazard categories reached 80 percent, highlighting the readiness of these models for implementation in safety decision-making pipelines.

Complementary to these structure-based approaches, integrated in vitro–in silico frameworks have gained traction for systemic toxicity and pharmacokinetic prediction, particularly in human-relevant exposure modeling. A bottom-up physiologically based biokinetic (PBK) model from *in vitro* assay data was constructed, and this model was shown to predict plasma concentrations and clearance values within a two-fold deviation from observed human pharmacokinetics (Chan et al., 2019). This level of precision supports its use in early human exposure assessments, replacing or supplementing animal pharmacokinetic studies. This was further exemplified by combining PBK modeling with quantitative in vitro-to-in vivo extrapolation (QIVIVE) to perform anti-androgenic risk assessments using the Dietary Comparator Ratio (DCR) framework (Van Tongeren et al., 2021). That method enabled accurate translation of *in vitro* effects into human-relevant exposure limits, addressing key regulatory challenges in endocrine disruption.

Moreover, transcriptomics-guided models have added another dimension to predictive toxicology. Gaussian process regression was utilized to develop a model predicting renal toxicity in rats based solely on human *in vitro* gene expression and molecular descriptors (Gardiner et al., 2020). With a reported R^2^ of 0.661, this model underscored the potential of human-based omics data to predict systemic outcomes traditionally assessed through animal experimentation. Mechanistic modeling has also been employed to simulate receptor-mediated and off-target toxicities. For instance, the integration of PBPK modeling, metabolic control analysis, and Petri net-based signaling frameworks was used to characterize complex tissue-specific responses across multiple administration routes (Leedale et al., 2018). This approach enabled simulation of dynamic feedback loops and emergent toxic effects in a systems biology context, moving beyond black-box predictions.

Computational fluid dynamics (CFD) has further enhanced prediction of drug behavior at the tissue level. A hybrid model integrated CFD with *in vitro* nanomaterial transport assays to simulate intracellular drug diffusion within live tissues (Price and Gesquiere, 2020). The results exhibited a high correlation (r = 0.861) between predicted and experimentally observed spatial drug distributions, validating the model’s applicability to real-time tissue pharmacokinetics.

**FIGURE 6:**
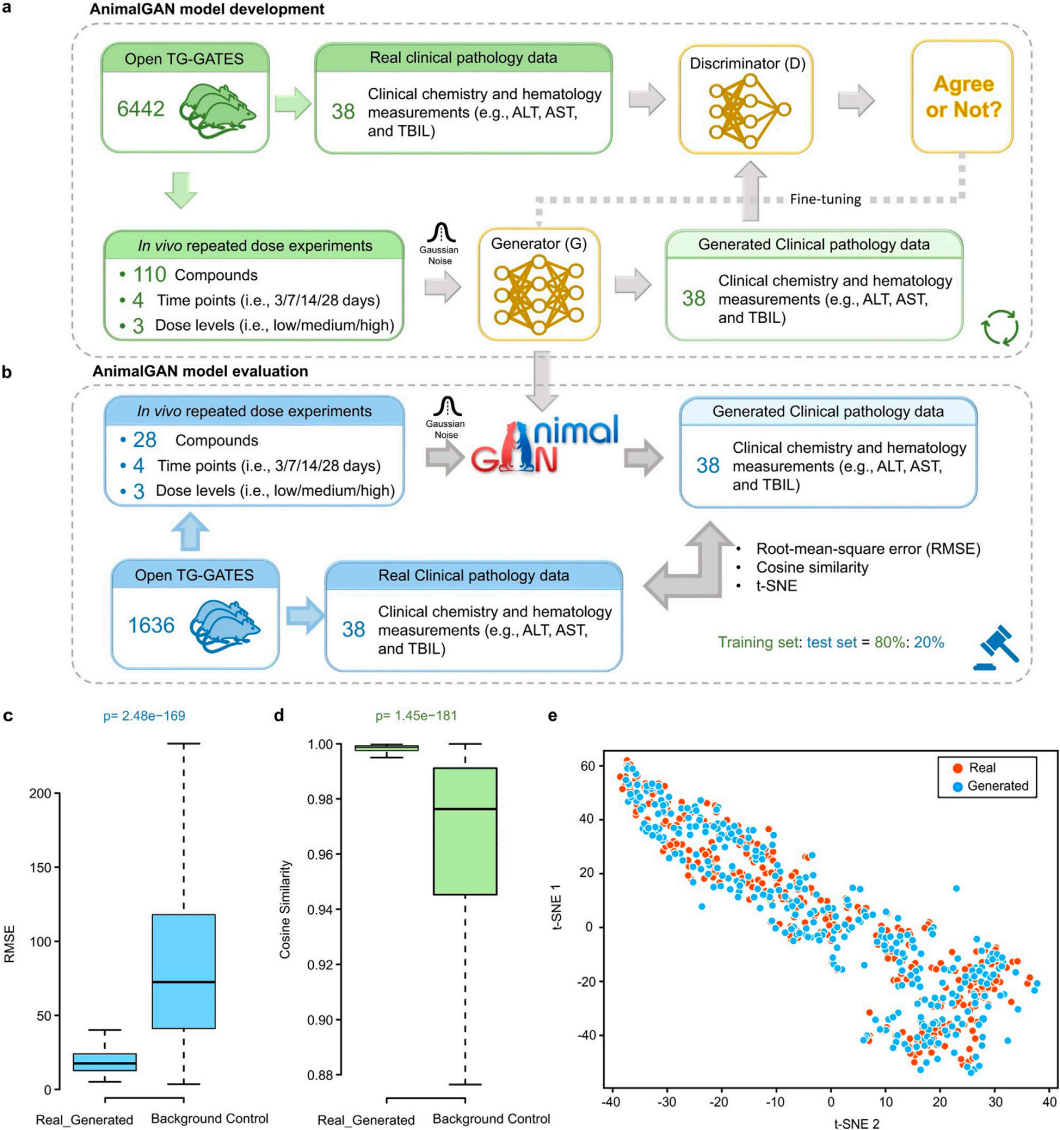
Overview of AnimalGAN model and workflow. **(a)** Schematic of model development using 80% of TG-GATEs data with compound- and dose-specific inputs. **(b)** Model evaluation on the remaining 20% of data. **(c,d)** Boxplots showing RMSE and cosine similarity between synthetic and real data. **(e)** t-SNE visualization comparing distributions of real and generated data. Taken from Chen et al. (2023) under a Creative Commons Attribution 4.0 International License.

**FIGURE 7:**
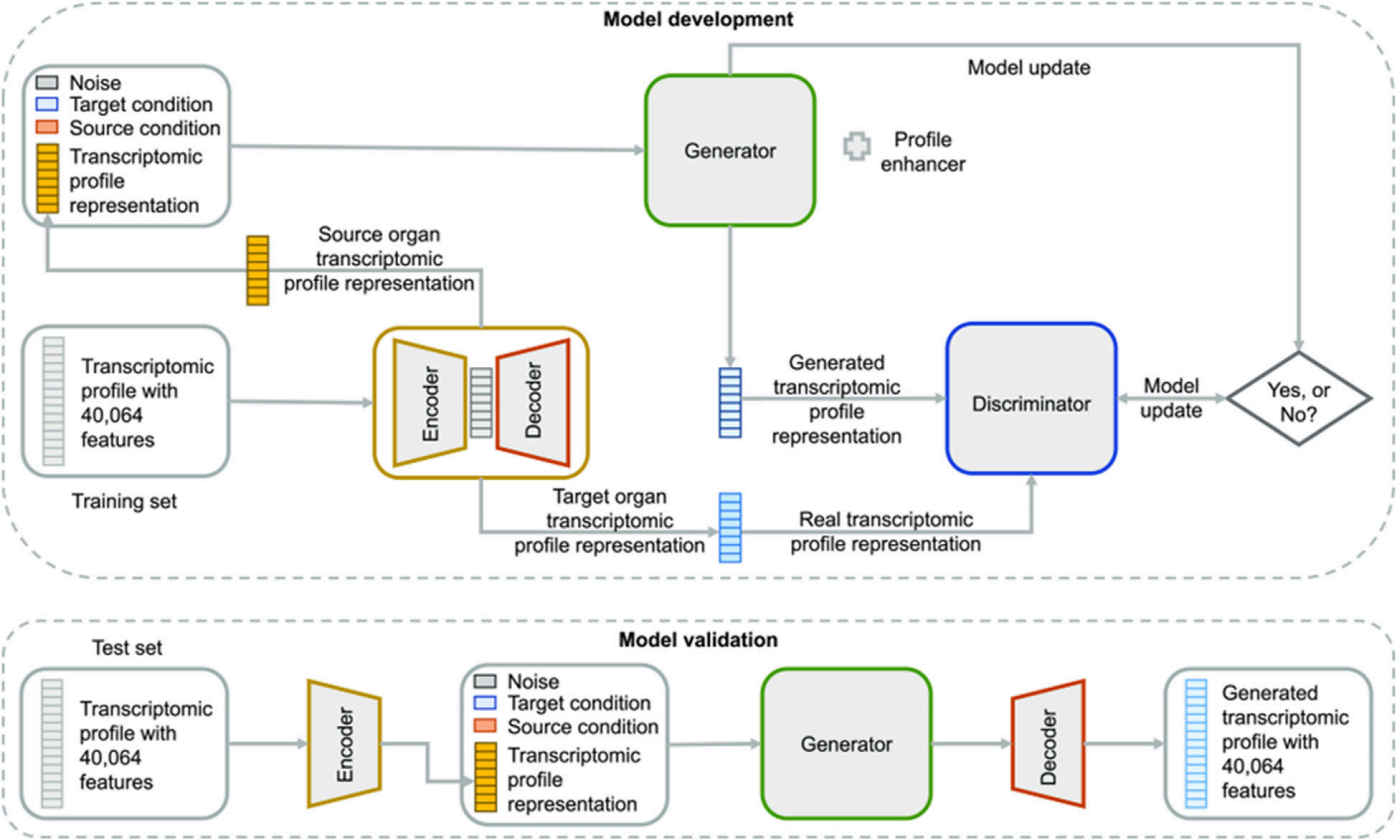
Overview of TransOrGAN model. The input transcriptomic profile is encoded and combined with source and target organ conditions plus noise. The generator produces a new profile compared to the target via a discriminator. The decoder reconstructs the full gene expression profile. Taken from (Li et al., 2023) under the Creative Commons License (CC-BY-NC-ND 4.0).

Most notably, recent innovations in generative modeling have opened new frontiers in non-animal-based toxicology. First was the introduction of AnimalGAN, a generative adversarial network trained on historical animal study data to predict clinical pathology parameters such as liver enzymes and hematological markers (Figure 6) (Chen et al., 2023). The model achieved 82.85 percent consistency with external DrugMatrix validation data and outperformed traditional QSAR models in predictive accuracy for key endpoints. In a complementary effort, TransOrGAN is a CycleGAN-based model capable of mapping transcriptomic data across biological contexts such as organ type, age, and sex in rats (Li et al., 2023). With cosine similarity scores exceeding 0.98 between synthetic and real gene expression profiles, TransOrGAN has demonstrated the feasibility of modeling complex biological variability *in silico*, thus supporting personalized toxicological predictions without the need for stratified animal studies (Figure 7).

### Infectious diseases

Computational modeling has emerged as a central component of infectious disease research, offering powerful tools to accelerate therapeutic discovery, elucidate host-pathogen interactions, and address the growing threat of antimicrobial resistance. The application of artificial intelligence (AI), machine learning (ML), and simulation-based platforms allows for the efficient analysis of large-scale biological datasets, modeling of complex systems, and prediction of drug-target interactions. These *in silico* approaches provide high-throughput capabilities for compound screening, mechanistic exploration, and simulation of infectious processes at both molecular and systems levels. Importantly, they also offer a more time-efficient, cost-effective, and ethically responsible alternative to traditional experimental methods, reducing reliance on animal testing and extensive laboratory-based assays.

Several recent studies have demonstrated the effectiveness of combining computational tools with experimental validation in the discovery of anti-infective agents. Biostimulated sesame sprout extracts were explored for their activity against *Leishmania mexicana* (Garduno-Felix et al., 2023). *In vitro* studies and infected macrophage models confirmed significant antileishmanial activity, while molecular docking revealed strong binding affinities, ranging from −8.4 to −9.6 kcal/mol, to essential parasite enzymes such as trypanothione reductase and pteridine reductase 1. Similarly, another study investigated a mastoparan–chitosan nanoconstruct that targets multidrug-resistant *Acinetobacter baumannii* and demonstrated therapeutic efficacy in a murine sepsis model (Hassan et al., 2021). Molecular dynamics simulations and docking analyses supported these findings by confirming the nanoconstruct’s membrane-targeting mechanism and structural stability during host-pathogen interaction.

In addition to studies that integrate experimental data, fully computational approaches have advanced early-stage therapeutic screening and host-pathogen interaction modeling. For example, there was the introduction of DeepLysin, a deep learning pipeline designed to mine bacteriophage genomes for antibacterial lysins (Zhang et al., 2024). Trained on curated lysin datasets, the model achieved precision and recall rates above 92 percent and enabled the identification of numerous previously uncharacterized lysin candidates. Next, ML algorithms were applied to predict phage-host interactions using protein domain composition data, attaining F1-scores above 90 percent (Leite et al., 2018). This model offers a scalable solution for identifying suitable phage therapy candidates, particularly in the context of antibiotic-resistant infections.

In another novel application of computational modeling, the area under the effect curve (AUEC) was introduced as a simulation-based index for evaluating anti-infective efficacy (Chen et al., 2022). Unlike conventional pharmacokinetic or pharmacodynamic metrics, AUEC incorporates both concentration and duration of drug exposure to better reflect pharmacological activity over time. Simulation studies showed that AUEC provided superior predictive performance for dose-response outcomes across species and infection models, enhancing the accuracy of cross-species dose extrapolation and supporting its use in translational modeling.

### Neurological disorders

In the field of neurological disorders, computational modeling has become an essential tool for accelerating therapeutic discovery, optimizing drug design, and reducing dependence on traditional animal-based research. The integration of molecular docking, ML, and microphysiological simulation platforms has enabled precise identification of bioactive compounds, prediction of pharmacokinetic behavior, and modeling of complex biological barriers such as the blood-brain barrier (BBB). These approaches are especially valuable in neurology, where drug delivery to the central nervous system and mechanistic validation of neuroprotective agents present considerable challenges.

One study employed molecular docking to identify phytoconstituents in the Ficus racemosa plant with strong binding affinity to AChE, β-secretase, and γ-secretase, targets implicated in the pathogenesis of Alzheimer’s disease (Rani et al., 2024). Docking scores for key compounds such as lupeol, racemosic acid, and quercetin ranged between −9.2 and −10.4 kcal/mol, with hydrogen bonding and hydrophobic interactions observed at the active sites of these enzymes. *In vivo* experiments in rats validated that these plant extracts exhibited neuroprotective effects, as evidenced by a 32 percent increase in SOD activity and a 27 percent increase in GSH levels relative to untreated controls. Histopathological evaluations of brain, liver, and kidney tissues revealed preserved cellular morphology, with no observable toxicity, supporting the compound’s safety profile. These results indicate that computational modeling was instrumental in guiding both compound selection and mechanistic interpretation.

Similarly, a ML scoring function to identify nonbioavailable substructures was developed with the goal of designing improved selective serotonin reuptake inhibitors (SSRIs) (Wang et al., 2023). Their model, trained on physicochemical descriptors and absorption data from over 150 serotonergic compounds, identified a specific substructure in vilazodone associated with low gastrointestinal permeability. After redesigning the molecule to replace the identified moiety, the modified compound demonstrated a 45 percent increase in oral bioavailability and a 1.8-fold increase in systemic plasma exposure in *in vivo* pharmacokinetic studies. This application of ML not only enhanced the drug’s performance but also eliminated the need for multiple rounds of trial-and-error synthesis.

Computation has also pioneered the advent of simulations to predict BBB interactions and optimize CNS drug permeability. A manufactured form of a microengineered physiological system–tissue barrier chip that successfully replicated key physiological features of the BBB was developed and validated (Kim et al., 2023). The chip incorporated human-derived endothelial cells, pericytes, and astrocytes within a dynamic microfluidic platform, achieving trans-endothelial electrical resistance (TEER) values above 200 Ω·cm^2^, consistent with human BBB *in vivo*. The model was tested with multiple CNS-penetrant and non-penetrant drugs, including caffeine, atenolol, and diazepam, and demonstrated a Pearson correlation coefficient (r = 0.91) between predicted and reported *in vivo* permeability values. Furthermore, the chip reproduced immune exclusion characteristics of the BBB, enabling its use in studies of neuroinflammation and immune cell trafficking in a human-relevant system.

### Ocular toxicity

Computational modeling has become an increasingly important tool in the field of ocular toxicology, offering reliable, scalable, and ethically responsible alternatives to traditional *in vivo* assays such as the Draize eye irritation test. Advances in ML, DL, and consensus modeling have enabled the development of predictive models that assess eye irritation and corrosion potential with a high degree of accuracy and mechanistic interpretability. These tools are particularly valuable in early-stage compound screening and regulatory decision-making, research stages where rapid and animal-free methods are urgently needed.

A comprehensive suite of ML and DL algorithms was applied to the largest binary-labeled ocular toxicity dataset compiled to date (Zhou et al., 2024). The approach included RF, XGBoost, and LightGBM models, as well as graph-based architectures such as Graph Convolutional Networks (GCNs) and Attentive Fingerprint (Attentive FP) networks. These graph-based models captured molecular structure–activity relationships more effectively than traditional fingerprint descriptors. The highest-performing model, Attentive FP, achieved an area under the ROC curve (AUC) of 0.915, indicating strong discriminatory power in classifying ocular toxicants. The study highlighted the capacity of deep learning frameworks to capture subtle topological features of molecules relevant to eye irritation, making them particularly well-suited for early-stage filtering of chemical libraries.

In a parallel effort, a series of ML models including random forest, gradient boosting, and consensus approaches was developed and augmented with an active learning protocol that dynamically prioritized data points for labeling based on model uncertainty (Di et al., 2022). This strategy led to the construction of a high-confidence training set and significantly improved predictive performance. Their model for serious eye damage achieved an accuracy of 0.972 and demonstrated excellent class separation, making it an effective tool for structure optimization and risk mitigation during compound design. The integration of active learning not only enhanced model efficiency but also minimized the number of experimental tests required for model refinement.

Further expanding the scope of ocular toxicity prediction, investigators compiled the largest expertly curated multiclass dataset for eye irritation and corrosion, encompassing multiple severity categories aligned with regulatory guidelines (Silva et al., 2021). They employed MuDRA-based quantitative structure–activity relationship (QSAR) modeling to develop classifiers capable of distinguishing among non-irritants and mild, moderate, and severe irritants. The irritation model achieved a balanced accuracy of 0.88, while the corrosion model reached 0.85, with both models maintaining high sensitivity and specificity. These models have strong potential for regulatory acceptance, particularly in screening programs and safety assessments under frameworks such as REACH and OECD guidelines.

### Oncology studies

Computational modeling has become a critical pillar in oncology research, offering robust tools to predict drug efficacy, toxicity, and resistance mechanisms with high precision while significantly reducing reliance on animal models and early-phase clinical trials. As cancer treatment moves increasingly toward personalized and mechanism-based strategies, *in silico* approaches are reshaping the landscape of translational oncology by facilitating rational drug design, virtual clinical trial simulations, and systems-level analysis of tumor behavior. These models harness structural, pharmacokinetic, pharmacodynamic, and systems biology data to guide therapeutic development and optimization across diverse cancer types.

One notable application of structure-guided drug design is demonstrated by the development of a proteolysis-targeting chimera (PROTAC) aimed at degrading the oncogenic transcription factor FOXM1, which is overexpressed in multiple malignancies and associated with poor prognosis (Luo et al., 2021). Using molecular docking and structural modeling, the authors designed and prioritized candidate PROTACs based on their predicted interaction with both the FOXM1 target and E3 ligase components. The lead compound, 17 days, exhibited a degradation concentration (DC_50_) of 1.96 μM *in vitro* and led to a 78.2 percent reduction in tumor growth in xenograft-bearing mice. Transcriptomic and protein-level analyses confirmed suppression of EMT-related markers such as N-cadherin and vimentin, validating the therapeutic mechanism. The computational design phase significantly reduced the experimental workload by narrowing the pool of viable candidates for *in vivo* validation, illustrating how docking and modeling can streamline the PROTAC development pipeline.

Complementing drug discovery efforts, a quantitative systems pharmacology (QSP) model was utilized to simulate hematological toxicity profiles of avadomide in patients with diffuse large B-cell lymphoma (DLBCL) (Abbiati et al., 2021). The model integrated pharmacokinetic and pharmacodynamic parameters with a detailed mathematical representation of the neutrophil life cycle using a system of ordinary differential equations. By generating virtual patient populations, the QSP framework predicted the frequency, severity, and recovery trajectories of grade 3–4 neutropenia under different dosing regimens. Simulations accurately mirrored clinical observations, enabling the identification of dose schedules that maintained efficacy while reducing hematologic adverse events. This approach underscores how *in silico* toxicity modeling can be employed in virtual dose-finding studies to optimize therapeutic windows, inform trial design, and mitigate risk before first-in-human testing.

Addressing tumor resistance mechanisms, patient-derived 3D tumor spheroid models were integrated with a Boolean-based *in silico* signaling network to study therapeutic resistance in KRAS-mutant non-small cell lung cancer (NSCLC) (Peindl et al., 2022). The computational model incorporated mutational profiles, signaling topology, and phenotypic states such as epithelial-to-mesenchymal transition (EMT) to simulate cellular responses to monotherapy and combination regimens. Simulations revealed variable sensitivity to KRASG12C inhibitors depending on EMT status and predicted that co-targeting aurora kinase A (AURKA) could overcome resistance in mesenchymal-like tumor subtypes. These predictions were subsequently validated using 3D organoid assays, confirming the synergy between KRAS and AURKA inhibition. This hybrid modeling strategy exemplifies how computational networks can contextualize drug response within tumor heterogeneity, guiding precision combination therapies in resistant cancers.

### Pregnancy-related drug kinetics

Understanding drug disposition during pregnancy remains a significant challenge due to the complex and dynamic physiological changes in the maternal–fetal environment, as well as the ethical constraints and logistical difficulties inherent in studying pregnant populations. Computational modeling is increasingly recognized as a critical tool to bridge these gaps by enabling the simulation of maternal–fetal pharmacokinetics (PK) and the prediction of placental drug transfer without reliance on invasive *in vivo* testing. Through the integration of machine learning (ML), quantitative structure–property relationships (QSPR), and physiologically-based pharmacokinetic (PBPK) modeling, researchers are now able to generate mechanistic insights and make data-driven predictions about fetal drug exposure and safety risks.

One example of such innovation is presented by the development of a chirality-sensitive QSPR framework specifically designed to predict the transfer of xenobiotics across the human placental barrier (Gomatam and Coutinho, 2024). Traditional QSAR models often lack stereochemical resolution, which limits their utility in modeling enantioselective drug transport. To address this, the authors applied the EVANS (EigenValue ANalySis) methodology to encode molecular stereochemistry into multidimensional descriptors, capturing both topological and 3D conformational features of chiral compounds. These descriptors were input into a suite of ML classifiers, including Naive Bayes, k-nearest neighbors (KNN), RF, LR, and SVM. The best-performing model, an SVM classifier, achieved a coefficient of determination (R^2^) of 0.75 for regression tasks, while the LR model classified compounds with 88 percent accuracy. This platform not only allowed for early-stage screening of environmental and pharmaceutical compounds but also provided interpretability by identifying key structural features associated with higher transplacental permeability, thereby improving risk stratification during drug development.

**FIGURE 8:**
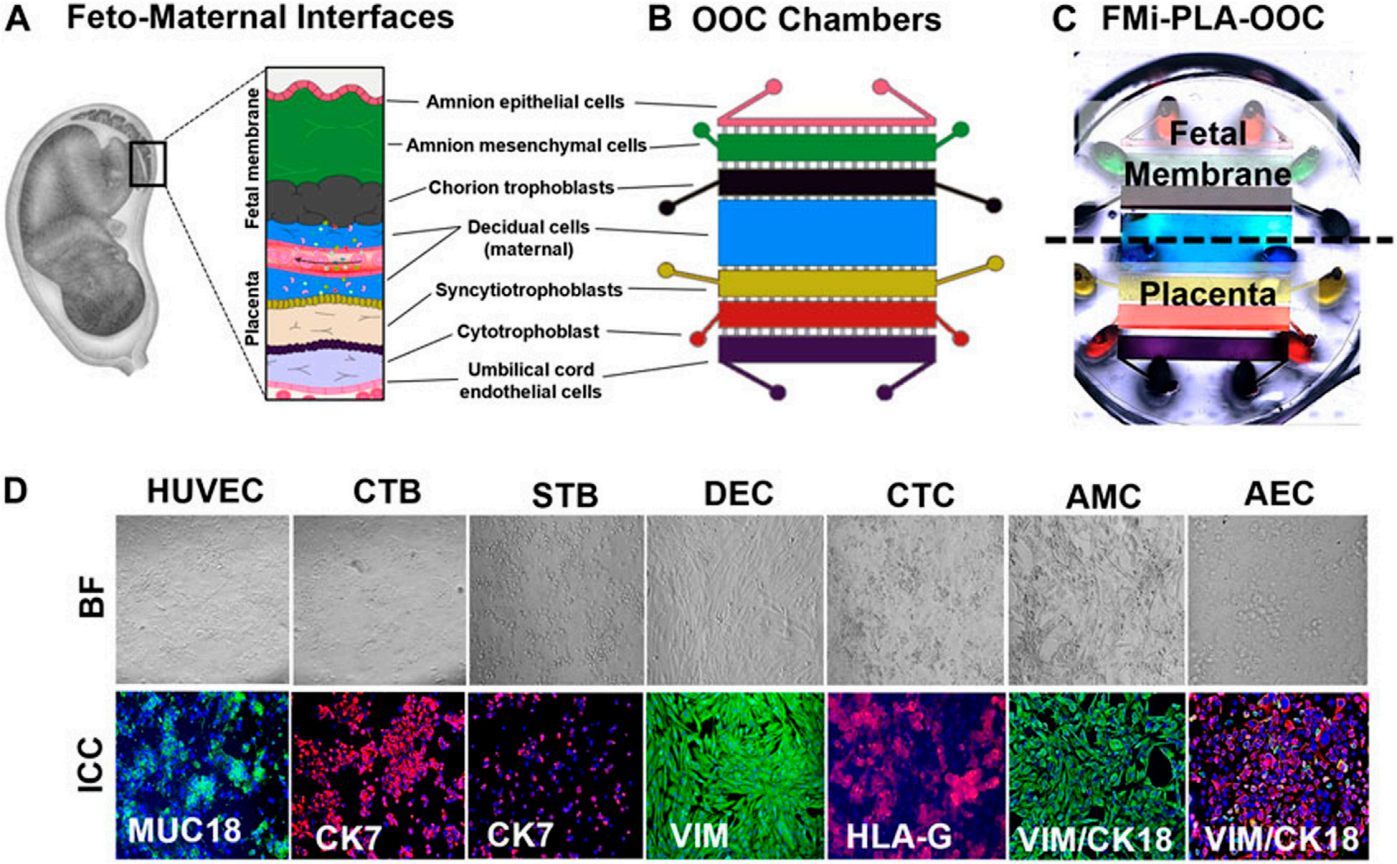
Development of the multi-organ Fetal Membrane-Placenta feto-maternal interface Organ-On-Chip (FMi-PLA-OOC) platform Development of the FMi-PLA-OOC platform. **(A)** Schematic of feto-maternal interface architecture. **(B)** Design of the microfluidic platform with layered cell chambers. **(C)** Device visualization with dye-filled chambers. **(D)** Validation of cellular identity and function using imaging and marker expression. Taken from Kammala et al. (2023) under the terms of the Creative Commons Attribution License (CC BY).

In a complementary approach, *in vitro* microfluidic experimentation was integrated with *in silico* PBPK modeling to develop a hybrid maternal–fetal drug disposition platform (Kammala et al., 2023). Their system, termed FMi-PLA-OOC (Fetal Membrane–Placental Organ-on-Chip), mimicked the structure and function of the human maternal-fetal interface using a microengineered co-culture of trophoblast, endothelial, and amniotic epithelial cells (Figure 8). The chip was used to measure real-time transfer of several test compounds across the placental barrier, and these empirical results were then incorporated into a PBPK framework that simulated maternal and fetal plasma concentration profiles. The *in silico* predictions from this model demonstrated strong concordance with *ex vivo* human placental perfusion data (R^2^ > 0.85) and significantly outperformed murine models that failed to replicate human transfer kinetics in over 30 percent of test cases. The integration of microphysiological systems with computational simulation allowed for precise control over experimental variables such as flow rate, hormone levels, and barrier integrity, which are often difficult to standardize in animal studies.

### Renal and urological disorders

The application of computational modeling in renal and urological research has gained increasing traction as an efficient and mechanistically grounded approach to accelerate drug discovery and reduce reliance on traditional trial-and-error methodologies. By integrating *in silico* predictions with *in vivo* validation, researchers are leveraging advanced computational techniques to improve the drug discovery process. Methods such as molecular docking, molecular dynamics simulations, and pharmacokinetic modeling are being used to identify, prioritize, and mechanistically assess novel therapeutic agents for conditions such as benign prostatic hypertrophy (BPH) and urolithiasis. These strategies not only improve the precision of early-stage compound selection but also enhance the translational relevance of preclinical findings.

In one study, sesamol, a phenolic compound derived from sesame oil, was evaluated for its potential use in treating BPH (Shah et al., 2021). The investigation began with molecular docking simulations targeting the androgen receptor (AR), a critical mediator in prostatic hyperplasia. Sesamol exhibited strong binding affinity (−8.4 kcal/mol) to the ligand-binding domain of AR, suggesting it could act as a functional modulator. Further computational analysis using ADME (absorption, distribution, metabolism, and excretion) profiling showed that sesamol possessed favorable drug-likeness properties and high gastrointestinal absorption potential. Molecular dynamics simulations confirmed the stability of the sesamol–AR complex over a 100-nanosecond trajectory, supporting the structural robustness of the interaction. These *in silico* results guided subsequent *in vivo* validation in testosterone-induced BPH rat models, where sesamol treatment significantly reduced prostate weight and prostatic index by over 30 percent while simultaneously restoring antioxidant enzyme levels, including catalase and superoxide dismutase, and reducing lipid peroxidation. This combination of predictive modeling and biological verification illustrates how computational tools can accelerate mechanistic screening and preclinical development.

Similarly, a high throughput docking pipeline to identify bioactive phytochemicals with potential anti-urolithiatic effects was utilized (Kale et al., 2024). A total of 38 plant-derived compounds were screened against 11 protein targets associated with urolithiasis, including calcium oxalate crystallization modulators, antioxidant enzymes, and renal epithelial damage mediators. Hesperidin, a citrus-derived flavonoid, emerged as the lead candidate, showing high binding affinities to key proteins such as Tamm–Horsfall protein, superoxide dismutase, and osteopontin, with docking scores ranging from −9.0 to −10.3 kcal/mol. Subsequent ADME-Tox modeling predicted low toxicity, high oral bioavailability, and minimal blood–brain barrier penetration, making hesperidin a suitable renal therapeutic candidate. This compound was then tested in both *Drosophila melanogaster* and murine models of sodium oxalate-induced urolithiasis. *In vivo* results showed a 46 percent reduction in renal crystal deposition, significant restoration of serum creatinine and urea levels, and histological evidence of reduced tubular injury. The inclusion of a non-mammalian model further demonstrated the compound’s conserved efficacy across species, enhancing its translational appeal.

### Skin sensitization

The prediction of skin sensitization has significantly advanced through the application of computational modeling, which now offers scientifically rigorous and ethically preferable alternatives to traditional *in vivo* assays such as the Local Lymph Node Assay (LLNA) and Guinea Pig Maximization Test (GPMT). In silico approaches have been particularly effective in early-stage hazard identification, compound screening, and regulatory decision-making. These models encompass a broad spectrum of methodologies, from data-driven ML and QSAR modeling to mechanistic immune simulations. Together, they represent a comprehensive strategy for capturing the complexity of skin sensitization pathways, including molecular initiation, immune activation, and T-cell response.

Recent studies have focused on refining ML-based and QSAR-integrated models to improve prediction accuracy, reduce data input complexity, and facilitate broad adoption. For example, HskinSensDS was developed as a decision-support system that combines multiple independent QSAR models through Dempster–Shafer theory, a mathematical framework for evidence synthesis (Wang et al., 2024). This ensemble model achieved classification accuracies of up to 88 percent and showed superior robustness across different chemical classes. The fusion of evidence from diverse QSAR predictors has allowed for greater certainty in decision-making, particularly when dealing with ambiguous or borderline compounds. Similarly, it was demonstrated that RF models trained exclusively on simple physicochemical descriptors such as logP, molecular weight, and hydrogen bond acceptors could reach F1-scores as high as 96 percent, making it possible to perform accurate skin sensitization screening using readily available molecular information (Im et al., 2023). These findings highlight the growing capability of lightweight, interpretable models in delivering high predictive performance with minimal data requirements.

Addressing domain-specific needs, PreS/MD was introduced as a deep learning model tailored for chemicals released from medical devices (Alves et al., 2022). Trained on historical GPMT data, the model achieved a balanced accuracy of 72 percent and a high negative predictive value (NPV = 0.82), indicating strong reliability in identifying non-sensitizers—a critical factor in regulatory screening where false positives can unnecessarily halt product development. The model was specifically designed to support ISO 10993-10 compliance, aligning with regulatory requirements for biocompatibility assessment. In a broader context, a standardized *in silico* framework for skin sensitization hazard identification was proposed, integrating expert-curated QSAR models with clear documentation for transparency and reproducibility (Johnson et al., 2020). The protocol emphasized mechanistic relevance, including model domains of applicability, decision thresholds, and validation parameters, thereby offering a blueprint for regulatory-aligned computational toxicology.

Beyond purely statistical models, efforts have been made to develop mechanistic simulations that mimic the biological processes underlying skin sensitization. An advanced computational pipeline was presented that combines molecular docking, T-cell epitope prediction, and agent-based modeling using the UISS-TOX immune simulation platform (Russo et al., 2024). This system modeled antigen processing, dendritic cell activation, and T-helper cell polarization to distinguish between Th1- and Th2-mediated responses, effectively simulating allergic phenotypes induced by different sensitizers. The model correctly classified a range of well-characterized sensitizers and non-sensitizers, demonstrating not only high predictive accuracy but also biological plausibility, which is critical for mechanistic understanding and regulatory acceptance.

## Discussion

This systematic review highlights the increasing utility of simulation models across therapeutic domains as scientifically robust alternatives to animal testing. The body of evidence synthesized from 45 studies illustrates that These models have demonstrated high predictive accuracy, improved translational fidelity, and ethical superiority in domains ranging from cardiovascular disease to toxicology.

Simulation models offer several distinct advantages over traditional animal models. Computational models, in particular, allow for high-throughput, scalable screening, while organ-on-chip systems replicate human tissue responses with greater fidelity than animal models. These tools reduce the translation gap that often leads to clinical trial failures, lack of reproducibility, and enable a move toward personalized medicine.

Numerous studies across therapeutic domains, including cardiovascular disease (Alauddin et al., 2024), diabetes (Paul et al., 2021; Lawal et al., 2022), oncology (Luo et al., 2021; Abbiati et al., 2021), and infectious diseases (Garduno-Felix et al., 2023; Zhang et al., 2024), have demonstrated that simulation models can reliably replicate pharmacodynamic and toxicological outcomes traditionally evaluated in animals. These models have enabled the identification of high-affinity drug-target interactions, forecasted adverse effects, and guided compound prioritization for experimental validation, often reducing the scope and necessity of animal-based studies.

The integration of artificial intelligence, machine learning, and deep learning techniques has been pivotal to this transformation. Predictive models such as VenomPred 2.0 (Di Stefano et al., 2024) and AnimalGAN (Chen et al., 2023) have achieved high accuracy in classifying toxicological endpoints, while models like AEGIS (Hartout et al., 2023) and TransOrGAN (Li et al., 2023) have expanded the capabilities of simulation into immunological modeling and transcriptomics translation, respectively. These findings support a paradigm shift toward data-driven drug discovery and toxicology.

Furthermore, the scalability and adaptability of these models enable them to simulate complex biological phenomena, including dynamic drug absorption and distribution, as illustrated by physiologically-based pharmacokinetic models (Chan et al., 2019; Van Tongeren et al., 2021) and computational fluid dynamics in tissue-level pharmacokinetics (Price and Gesquiere, 2020). These techniques offer advantages in reproducibility and control that are difficult to achieve in animal studies.

Nonetheless, significant barriers to the full integration of simulation models into regulatory and translational workflows remain. The key challenges among these are the standardization of modeling frameworks, validation against clinical or experimental benchmarks, and the need for transparent reporting practices (Sharma et al., 2023; Bercu et al., 2021). While several studies have achieved high performance metrics, such as area under the curve (AUC) values exceeding 0.90 (Feng et al., 2021; Zhou et al., 2024), the lack of uniform validation protocols complicates cross-study comparisons and regulatory acceptance.

Encouragingly, recent regulatory movements have begun to support non-animal methodologies. The U.S. Food and Drug Administration’s Predictive Toxicology Roadmap and the ISTAND initiative emphasize the inclusion of advanced simulation tools for regulatory submissions (FDA, 2024), while the FDA Modernization Act 2.0 explicitly permits alternatives such as organ-on-chip systems and *in silico* models (Kaplan et al., 2024). These developments signal a growing institutional commitment to non-animal research innovation.

The reviewed literature also underscores the promise of hybrid systems, such as the integration of microengineered organ-on-chip platforms with simulation models in maternal–fetal pharmacokinetics (Kammala et al., 2023) and neurotoxicity (Kim et al., 2023). These systems, capable of replicating human barrier functions and immune interactions, add physiological relevance that bridges the gap between purely computational models and *in vivo* complexity.

In conclusion, simulation models offer a viable and scientifically robust alternative to animal testing, with applications spanning early-stage compound screening to complex systems-level analyses. While regulatory validation and methodological harmonization are ongoing challenges, the convergence of computational, experimental, and engineering disciplines positions simulation models to play a central role in the future of ethical, personalized, and efficient biomedical research.

## Challenges with the simulation model

Despite the valuable insights provided by the simulation model, several limitations must be acknowledged. Primarily, the model operates under idealized conditions and does not incorporate real-world variables such as environmental fluctuations, batch-to-batch variability in biological systems, or human error during experimental procedures. These omissions may affect the external validity of the predictions. Additionally, the model’s predictive accuracy may diminish over extended time periods due to the increasing complexity and nonlinearity of biological and pharmacological interactions. Furthermore, the model relies heavily on input data quality and assumptions embedded in its architecture, which may not fully capture the nuances of *in vivo* physiology. These limitations highlight the need for continued validation and refinement using diverse datasets and experimental corroboration to enhance model robustness and translational relevance.

## Limitations

While this systematic review provides a broad and in-depth synthesis of current simulation model applications in drug development and biomedical research, several limitations should be acknowledged.

A major challenge encountered was the heterogeneity across studies in terms of simulation design, input data sources, validation protocols, and outcome metrics. Such variability limited this review’s ability to perform direct comparisons or derive standardized benchmarks of model performance. Differences in endpoints, ranging from toxicity classification to pharmacokinetic modeling, along with inconsistent use of external validation, constrain the generalizability of findings and underscore the need for harmonized evaluation frameworks.

While many studies reported strong internal validation metrics, relatively few demonstrated performance across independent datasets or real-world clinical scenarios. This lack of external benchmarking raises concerns about potential model overfitting and limits confidence in broader translational applicability. In particular, models trained on narrowly scoped or proprietary datasets may perform well *in silico* but fail to generalize across different biological systems, populations, or experimental conditions.

Reproducibility remains another critical concern. In several cases, access to simulation code, training data, or modeling assumptions was limited, making independent replication difficult. As computational modeling becomes more integrated into regulatory science and preclinical workflows, transparent reporting and open-source dissemination will be essential to ensure credibility and facilitate collaborative refinement.

Although the review focused on simulation models as potential alternatives to animal experimentation, it is important to acknowledge that full replacement remains context-dependent. In complex domains—such as immunotoxicology, developmental biology, and systems-level physiology—simulation models often complement rather than replace animal testing, providing mechanistic insights or screening capacity that inform but do not yet substitute for *in vivo* studies.

Finally, the potential for publication bias should be considered. Studies with favorable or innovative outcomes are more likely to appear in the published literature, possibly overestimating the maturity and general readiness of simulation tools. This highlights the need for balanced reporting that also reflects null or underperforming findings, which are critical for identifying limitations and advancing model refinement.

Despite these constraints, the collective evidence supports the growing utility of simulation models in accelerating therapeutic discovery, reducing reliance on animal models, and improving the mechanistic interpretability of preclinical data. Future efforts focused on standardization, cross-validation, and open science practices will be key to addressing these limitations and realizing the full potential of computational approaches in biomedical research.

## Conclusion and future perspectives

The accelerating development of simulation models in biomedical research marks a pivotal shift in the methodological landscape of drug discovery and safety assessment. These technologies, spanning *in silico* prediction, mechanistic modeling, and hybrid organ-on-chip integration, are not merely supplements to existing paradigms but are emerging as foundational components of a new computational preclinical ecosystem. This transition is underpinned by advances in algorithmic design, data integration, and computational power, which together enable nuanced representations of human biology previously inaccessible to traditional experimental frameworks.

Importantly, the value of simulation models extends beyond reproducing established biological processes. Their true potential lies in the ability to uncover causal mechanisms and generate predictive insights that reach beyond the limitations of empirical data alone. The ability to simulate virtual populations, explore parameter uncertainty, and test mechanistic hypotheses at scale opens a new frontier in hypothesis-driven modeling that is both ethically aligned and scientifically ambitious. As these systems continue to mature, they offer a pathway toward modeling emergent phenomena such as inter-individual variability, off-target effects, and longitudinal treatment responses in ways that animal models and conventional assays cannot capture.

Future progress will depend on targeted investment in three key domains. First, the development of standardized validation frameworks capable of benchmarking model performance across datasets, disease contexts, and regulatory endpoints will be critical for translational credibility. Second, sustained emphasis on interoperability and data transparency—including open-access codebases, modular architectures, and community-curated training datasets—will be necessary to facilitate replication and innovation. Third, greater interdisciplinary collaboration between computational scientists, experimental biologists, and regulatory stakeholders will be essential to ensure that emerging models are not only technically robust, but also aligned with evolving ethical, clinical, and regulatory priorities.

Ultimately, the adoption of simulation models should not be viewed as a replacement for current methodologies but as a redefinition of what constitutes preclinical evidence. By reimagining the early phases of biomedical research through the lens of computational modeling, the field stands poised to accelerate therapeutic innovation, enhance reproducibility, and transition toward a more human-relevant, data-driven framework for drug development.

## Data Availability

The original contributions presented in the study are included in the article/Supplementary Material, further inquiries can be directed to the corresponding authors.
