# Supplementary material for: Exploring the potential of computer simulation models in drug testing and biomedical research: a systematic review
Source: Front Pharmacol. 2025 Sep 24;16:1644907. doi: 10.3389/fphar.2025.1644907 (PMC12504290; doi:10.3389/fphar.2025.1644907)
Supplement: Supplementary file 1 [file Table1.pdf]

Supplementary Table 1: A Summary of Included Studies

Reference	Study Type	Disease Category	Animal Model	Objective	Computational Component	In Vitro/In Vivo/In Silico	Outcomes
Abbiati 2021	Diagnostic Test Accuracy	Oncology Studies	None	To develop a quantitative systems pharmacology (QSP) model of avadomide-induced neutropenia that enables virtual simulation of dose and schedule scenarios in a disease-specific virtual patient population, with the aim of guiding dose optimization and toxicity management in clinical trials.	Ordinary Differential Equation based Quantitative Systems Pharmacology model	In Silico, In Vitro	● The QSP model successfully predicted incidence, duration, and recovery of high-grade neutropenia in a virtual population of DLBCL patients.<br>● Identified dosing schedules and drug exposures that minimized prolonged grade 3/4 neutropenia while enabling recovery, such as the 7/14 and 21/28 schedules.
Adnan 2020	Quasi-Experimental	GI and Hepatic Disorders	Mice	Investigation of the pharmacological activities of Cuscuta reflexa leaves for neuro-pharmacological, anti-nociceptive, and antidiarrheal activities.	Molecular docking, ADMET prediction,	In Silico, In Vivo	● Methanolic extract of Cuscuta reflexa (MECR) was shown to have significant anxiolytic, antidepressant, anti-nociceptive, and antidiarrheal activities.<br>● In-silico studies revealed that isolated compounds displayed favorable

								binding affinities to target receptors.
Alam 2021	Quasi-Experimental	GI and Hepatic Disorders	Mice	To investigate the antidiarrheal, antimicrobial and antioxidant potential of the methanol soluble extract of Colocasia gigantea plant	Molecular docking, ADMET prediction	In Silico, In Vivo, In Vitro	• Colocasia gigantea is shown to be a promising therapeutic for antidiarrheal and mild antimicrobial treatment.<br>• Computational analysis revealed optimistic binding affinity to specific proteins of the compound.
Alauddin 2024	Quasi-Experimental	Cardiovascular Disorders	Rats	Determine the bioinformatics of identified soy peptides and their effect on reducing systolic blood pressure (BP) in spontaneously hypertensive rats (SHR)	Molecular docking, Toxicity prediction	In Silico, In Vivo	• Molecular docking simulations demonstrated that the FFYY peptide exhibited the strongest binding affinity to the ACE active site.<br>• In vivo findings showed this specific peptide significantly reduced blood pressure and ACE activity in spontaneously hypertensive rats without adverse effects.

Albrecht 2019	Diagnostic Test Accuracy	GI and Hepatic Disorders	None	To introduce a vitro/in silico method to predict the risk of human hepatotoxicity and drug-induced liver injury associated with oral doses and blood concentrations of compounds	SVM, PBKP model	In Silico, In Vitro	● This novel computational method for DILI based on in vitro test results reached a cross-validated sensitivity, specificity and accuracy for hepatotoxicity prediction of 100, 88 and 93% respectively.
Alves 2022	Diagnostic Test Accuracy	Skin Sensitization	None	To develop and validate a publicly accessible in silico tool, PreS/MD, that predicts the skin sensitization potential of chemicals released from medical devices using historical GPMT data.	QSAR Models, SVM, RF, LightGBM, DL	In Silico	● The DL model demonstrated reliable identification of non-sensitizers.<br>● It achieved a balanced accuracy of 72%, NPV of 0.82 and PPV of 0.6.
Ambe 2018	Diagnostic Test Accuracy	GI and Hepatic Disorders	None	To develop predictive classification models of hepatocellular hypertrophy based on molecular descriptors of hypertrophy in rats.	Deep learning, RF, SVM	In Silico	● The predictive model for hepatocellular hypertrophy of chemicals reached peak accuracy of 0.76, sensitivity of 0.90, and AUC of 0.81.

Bercu 2021	Diagnostic Test Accuracy	General Toxicity and Pharmacokinetic Prediction	None	To evaluate whether existing (Q)SAR models for acute oral toxicity, provided by Leadscope, are sufficiently reliable for regulatory use in GHS classification and labelling across chemical sectors.	QSAR (expert rule-based and statistical-based)	In Silico	● The consensus model correctly or conservatively predicted GHS categories for ~95% of chemicals.<br>● Balanced accuracy assessment showed 80.4% correct or more conservative classification across categories.
Chan 2019	Diagnostic Test Accuracy	General Toxicity and Pharmacokinetic Prediction	None	Develop and validate bottom-up physiologically-based biokinetic (PBK) modeling as an alternative to animal testing for deriving biokinetic data	PBK Modeling	In Silico, In Vitro	● Bottom-up PBK models could predict systemic exposure, maximum plasma concentration, plasma clearance, and time to reach Cmax within two-fold of the observed in-vitro data.
Chen 2022	Diagnostic Test Accuracy	Infectious Diseases	None	To assess the area under the effect curve as an alternative pharmacokinetic/pharmacodynamic index for predicting in vivo efficacy of anti-infectives, and to evaluate its potential as a more robust, cross-species translational tool compared to conventional indices like AUC, Cmax, and	PK/PD simulations	In Silico	● Across 17 simulation scenarios, AUEC consistently outperformed conventional PK/PD indices, showing strong correlation with in vivo efficacy (R^2 = 0.76–0.98) and less sensitivity to dosing interval.

				TEC50.			
Chen 2023	Diagnostic Test Accuracy	General Toxicity and Pharmacokinetic Prediction	None	The objective was to create a model that could generate synthetic animal data by learning from legacy animal study results, thus reducing the need for animal testing.	GAN	In Silico	• AnimalGAN outperformed all conventional QSARs in prediction of clinical pathology measurements and reached 82.85% consistency in external validation compared to DrugMatrix.
Di 2022	Diagnostic Test Accuracy	Ocular Toxicity	None	To develop computational models to predict the potential of compounds to cause serious eye damage (ED) or eye irritation (EI)	RF, TE, GBT, RBF, Consensus Modeling	In Silico	• The active learning model reached an accuracy of 0.972 and Matthews Correlation Coefficient (MCC) of 0.942.
Di Stefano 2024	Diagnostic Test Accuracy	General Toxicity and Pharmacokinetic Prediction	None	To develop VenomPred 2.0, an enhanced machine learning platform capable of predicting multiple toxicity endpoints of small molecules, including androgenicity, skin and eye irritation, acute oral toxicity, carcinogenicity, mutagenicity,	RF, SVM, KNN, multilayer perceptron	In Silico	• VenomPred 2.0 achieved high predictive accuracy, with Matthews Correlation Coefficient (MCC) values up to 0.94 for androgenicity and >0.50 for most endpoints.<br>• The integration of SHAP-based structural interpretation enabled the identification of likely toxicophores.

				estrogenicity, and hepatotoxicity, using interpretable AI strategies.			
Feng 2021	Diagnostic Test Accuracy	General Toxicity and Pharmacokinetic Prediction	None	To build and validate ensemble machine learning models using molecular fingerprints for the accurate prediction of reproductive toxicity in chemical compounds, with the aim of supporting non-animal-based early risk assessment.	SVM, RF, XGBoost	In Silico	• The best-performing model achieved strong predictive performance, with an external cross-validated AUC of 0.920.
Gardiner 2020	Diagnostic Test Accuracy	General Toxicity and Pharmacokinetic Prediction	None	To develop a machine learning model that can predict drug-induced renal toxicity in rats, using inexpensive human in vitro transcriptomic data and chemical structure features, thereby reducing reliance on animal testing.	Linear Regression, RF, SVR, XGBoost, Gradient Boosting, GP, KNN, LightGBM	In Silico, In Vitro	• The best-performing model (Gaussian Process with hierarchical t-SVD) predicted rat BUN levels with high accuracy (R^2 = 0.661, RMSE = 1.528 mg/dL) and low uncertainty.

Garduño-Félix 2023	Quasi-Experimental	Infectious Diseases	None	To evaluate the leishmanicidal potential of phenolic compounds extracted from Bacillus clausii-biostimulated black sesame sprouts, using a combination of in silico docking and in vitro biological assays.	Molecular Docking	In Silico, In Vitro	• The B. clausii-biostimulated sesame sprout extracts showed potent leishmanicidal activity, with IC_{50} = 0.08 mg GAE/mL (extracts) and 6.42 µM (apigenin).<br>• Significantly reduced NO production and parasite burden in infected macrophages.
Gomatam 2024	Diagnostic Test Accuracy	Pregnancy-Related Drug Kinetics	None	To develop and validate chirality-sensitive quantitative structure-property relationship (QSPR) models using the EVANS methodology and machine learning to predict chemical transfer across the human placental barrier.	EigenValue ANalySis (EVANS), Naive Bayes, KNN, RF, LR, SVM	In Silico	• The best performing models, SVM and LR, reached a coefficient of determination of 0.75 and accuracy of 0.88 respectively.
Hartout 2023	Diagnostic Test Accuracy	Diabetes and Related Disorders	None	To develop and validate a transformer-based protein language model (AEGIS) capable of predicting peptide presentation by MHC class II molecules across humans and mice.	RNN, compact transformer	In Silico	• The AEGIS model achieved AUC > 0.95 on most human datasets.<br>• AUC = 0.88 on the NOD mouse I-Ag7 dataset.<br>• Outperforming prior models, AEGIS represents the first accurate transformer-based model for predicting MHC-II peptide presentation.

Hassan 2021	Quasi-Experimental	Infectious Diseases	Mice	To design and characterize a mastoparan-loaded chitosan nanoconstruct and evaluate its antibacterial activity against clinical MDR A. baumannii strains using in silico, in vitro, and in vivo methodologies.	Molecular dynamic simulations	In Silico, In Vivo, In Vitro	• The Mast-Cs NC significantly reduced bacterial colony counts in mice compared to chitosan and mastoparan alone ($p < 0.0001$).<br>• Demonstrated excellent biocompatibility and cytocompatibility.
Im 2023	Diagnostic Test Accuracy	Skin Sensitization	None	To develop a machine-learning model for predicting skin sensitization using readily available physicochemical properties, with the goal of offering a quicker and easier screening method for chemical sensitization hazards	RF, SVM, QSAR	In Silico	• Presentation of a RF model for skin sensitization that outperformed SVMs and QSARs across classification schemes.<br>• RF models reached 96% and 82% F1 for binary and ternary classification respectively.

Johnson 2020	Diagnostic Test Accuracy	Skin Sensitization	None	To develop and present a standardized hazard assessment framework for predicting skin sensitization in humans using in silico models	QSAR Models	In Silico	● Presentation of a reproducible and expert-reviewed in silico hazard assessment protocol for predicting skin sensitization.<br>● Emphasizes transparency, mechanistic relevance, and alignment with regulatory frameworks.
Kale 2024	Quasi-Experimental	Renal and Urological Diseases	Mice, Fruit Flies	To evaluate the anti-urolithiatic activity of hesperidin using a combined in silico screening and in vivo validation in fruit fly and murine models.	Molecular Docking	In Silico, In Vivo	● Hesperidin demonstrated high binding affinity in molecular docking simulations.<br>● In vivo experiments significantly reduced renal crystal formation and normalized kidney function biomarkers, showing superior efficacy over the standard treatment Cystone.

Kamiya 2021	Diagnostic Test Accuracy	GI and Hepatic Disorders	None	To develop a tool for prediction of both influx and efflux permeability across intestinal cell monolayers for chemicals, allowing accurate estimation of oral absorption of a diverse range of chemicals and drugs in humans.	LightGBM, linear regression analysis	In Silico, In Vitro	• The implemented machine-learning system exhibited strong predictive power of intestinal permeability coefficients across a diverse range of chemicals<br>• The LightGBM model displayed an influx prediction correlation of 0.84 ($p < 0.001$) and an efflux prediction correlation of 0.83 ($p < 0.001$).
Kammala 2023	Diagnostic Test Accuracy	Pregnancy-Related Drug Kinetics	None	To develop and validate a combined microfluidic organ-on-chip and in silico simulation platform to study drug pharmacokinetics during pregnancy as an alternative to animal testing.	Microfluidic Fetal Membrane-Placental Feto-Maternal Interface On-Chip, ADMET prediction, PBPK modeling	In Silico, In Vitro	• The FMi-PLA-OOC chip and PBPK simulation yielded pravastatin transfer rates consistent with human placenta perfusion studies and closely aligned with clinical data.<br>• Outperformed mouse models that showed supraphysiologic transfer.
Kim 2023	Diagnostic Test Accuracy	Neurological Disorders	None	To develop and validate a standardized, reproducible blood–brain barrier organ-on-chip model (MEPS-TBC) that incorporates key cellular components and mimics physiological conditions for use in	Computational Fluid Dynamics	In Silico, In Vitro	• The MEPS-TBC chip successfully replicated key physiological features of the BBB, including 2× higher TEER values and significantly reduced dextran permeability compared to endothelial-only models.

				drug screening and barrier function studies.			
Lawal 2022	Quasi-Experimental	Diabetes and Related Disorders	Rats	To evaluate the hypoglycemic, antioxidant, and anti-inflammatory properties of Azanza garckeana methanolic extract using in vitro, in vivo, and in silico approaches, and to characterize its major bioactive compounds via mass spectroscopy and pharmacoinformatics.	ADMET, Molecular docking	In Silico, In Vivo, In Vitro	● Azanza garckeana extract significantly improved blood glucose control, insulin levels, antioxidant enzyme activity, and reduced inflammation and oxidative stress in diabetic rats.<br>● Molecular docking also showed strong binding affinity.
Leedale 2018	Diagnostic Test Accuracy	General Toxicity and Pharmacokinetic Prediction	None	To develop a combined in vitro/in silico framework for identifying off-target receptor-mediated toxicity, with the aim of reducing reliance on animal testing and improving mechanistic understanding of adverse drug reactions.	Petri Net-Based Signaling Model, Metabolic Control Analysis, PBPK	In Silico, In Vitro	● The combined framework successfully predicted off-target receptor activation and downstream signaling dynamics, identifying tissue-specific receptor responses for different administration routes (IV vs. oral).<br>● It demonstrated the ability to simulate potential systemic toxicity profiles and guide targeted follow-up testing

Leite 2018	Diagnostic Test Accuracy	Infectious Diseases	None	To develop machine learning models capable of predicting phage-bacterium interactions using features extracted from genomic data, with the goal of accelerating phage therapy development by identifying potential host-pathogen pairs without laboratory infection assays.	KNN, RF, SVM, ANN	In Silico	● The optimized artificial neural network (ANN) model achieved accuracy > 85.7%, f1-score >86.2%, sensitivity > 85.4% and specificity > 86.3% across 4 independent data sets
Li 2023	Diagnostic Test Accuracy	General Toxicity and Pharmacokinetic Prediction	None	To develop and validate TransOrGAN, a generative adversarial network (GAN)-based model that can translate transcriptomic profiles between different organs, ages, and sexes in rats, offering a scalable, ethical alternative to predict toxicity profiles without generating new animal data.	GAN	In Silico	● TransOrGAN generated transcriptomic profiles with high fidelity, achieving cosine similarity scores >0.98 for most organ, sex, and age comparisons.<br>● Demonstrated strong potential to enable toxicity inference across biological variables (e.g., predicting female or aged responses from male, young data).

Luo 2021	Quasi-Experimental	Oncology Studies	Mice	To design and validate a celebron-recruiting proteolysis-targeting chimera capable of targeting the FOXM1 oncoprotein for proteasomal degradation, offering an alternative to conventional transcription factor inhibition in cancer therapy.	Molecular Docking	In Silico, In Vivo, In Vitro	● The lead compound 17d achieved potent FOXM1 degradation in TNBC cells with a DC_{50} of 1.96 µM.<br>● Inhibited tumor growth by 78.2% in vivo.<br>● Significantly suppressed EMT-related gene expression and cancer cell invasiveness.
Mohanty 2019	Quasi-Experimental	Diabetes and Related Disorders	Rats	To evaluate the Dipeptidyl peptidase-IV (DPP-IV) inhibitory activity of Terminalia arjuna and to compare its efficacy with standard, synthetic DPP-IV inhibitors through a multi-modal assessment (in silico, in vitro, in vivo)	Molecular docking	In Silico, In Vivo, In Vitro	● T. arjuna extract showed significant DPP-IV inhibition (86.4%).<br>● It reduced HbA1c a in diabetic rats.<br>● Key constituents exhibited superior binding energy to DPP-IV compared to standard treatments.
Paul 2021	Quasi-Experimental	Diabetes and Related Disorders	Rats	To investigate the potential of supercritical carbon dioxide (SC-CO2) extracts of small cardamom (SC) and yellow mustard (YM) seeds as treatment for type 2 diabetes in streptozotocin-induced Wistar albino	Molecular docking, computational modeling (iHOMA2)	In Silico, In Vivo	● In vitro findings found 31.49% FBG reduction with SC extract and 32.28% reduction with YM extract.<br>● In iHOMA2 predictive modeling, insulin sensitivity increased by 35.68% for SC extract and 33.08% for YM extract.

				rats by in vivo and in silico methods.			
Peindl 2022	Diagnostic Test Accuracy	Oncology Studies	None	To assess the relationship between epithelial-to-mesenchymal transition (EMT), stemness, and drug resistance in NSCLC using a biologically relevant 3D tumor model and simulate drug responses through an integrated in silico signaling network, particularly focusing on KRAS-mutant lung cancer.	Boolean-based Dynamic Signaling Network Model	In Silico, In Vitro	● The combined model correctly predicted differential sensitivity to KRASG12C inhibition between H358 and HCC44 cells<br>● in silico simulations identified aurora kinase A (AURKA) inhibition as an effective combinatorial strategy in resistant KRAS-mutant cell lines with high EMT and c-MYC expression.
Price 2020	Diagnostic Test Accuracy	General Toxicity and Pharmacokinetic Prediction	None	To introduce a combination in vitro assay and computational fluid dynamic model to predict drug biodistribution within single cells of live animal tissue without the need for animal studies	Computational modeling	In Silico, In Vitro	● Successfully demonstrated the utility of computational modeling to allow prediction of biodistribution in animals for nano-materials, reaching a correlation of 0.861 between model-predicted and observed averages.<br>● ~50% of simulated tissue-

							level nanomaterial (NM) uptake values fell within <2-fold error of observed in vivo data while ~83% fell within <3-fold error.
Rani 2024	Quasi-Experimental	Neurological Disorders	Rats	To evaluate the pharmacognostic, pharmacological, and toxicological profile of ethanolic fruit and leaf extracts of Ficus racemosa, and to assess their therapeutic potential in Alzheimer's disease by targeting AChE, β-secretase, and γ-secretase enzymes.	Molecular Docking	In Silico, In Vivo, In Vitro	● Identification of several non-toxic phytoconstituents with strong in silico binding affinities to AChE, β-secretase, and γ-secretase (e.g., linoleic acid, behenic alcohol).<br>● Docking scores for these top compounds exceeded −9.2 kcal/mol for AChE and −7.3 kcal/mol for β-secretase, with no observed toxicity in mice.
Russo 2024	Diagnostic Test Accuracy	Skin Sensitization	None	To develop a bioinformatics-driven protocol that integrates molecular docking, epitope prediction, and agent-based modeling to forecast skin or respiratory allergic reactions to chemical sensitizers.	Molecular Docking, AlphaFold, Epitope Prediction (IEDB), Immune Response Simulation (UISS-TOX)	In SIlico	● The UISS-TOX model successfully differentiated between Th1-driven (skin) and Th2-driven (respiratory) allergic responses for known sensitizers

Shah 2021	Quasi-Experimental	Renal and Urological Diseases	Rats	To investigate the potential of sesamol and its derivatives as therapeutic agents for benign prostatic hypertrophy, using molecular docking, ADME analysis, molecular dynamics simulations, and rat BPH models.	ADMET, Molecular Docking, Molecular Dynamics Simulations	In Silico, In Vivo	● BS extract significantly reduced nitric oxide (NO) production by ~45–50% in infected macrophages ($p < 0.05$).<br>● Significant reduction in parasite burden was observed in treated macrophages ($P < 0.05$), comparable to amphotericin B.<br>● Key phenolic compounds (e.g., sesaminol 2-O-triglucoside, pinoresinol dihexoside, apigenin) showed binding energies higher than those of enzyme substrates and amphotericin B in many cases.
Sharma 2023	Diagnostic Test Accuracy	General Toxicity and Pharmacokinetic Prediction	None	To develop a multi-task deep learning model capable of simultaneously predicting in vitro, in vivo, and clinical toxicity, while enhancing model interpretability using contrastive explanations.	Multi-task Deep NNs, Single-Task Deep NNs, Contrastive Explanations Method	In Silico	● The multi-task model using SMILES embeddings significantly improved clinical toxicity prediction (AUC-ROC ≈ 0.99, balanced accuracy ≈ 0.96), outperforming baseline models.<br>● The contrastive explanation method (CEM) successfully identified and verified toxicophores and non-toxic substructures.

Silva 2021	Diagnostic Test Accuracy	Ocular Toxicity	None	Consolidate the largest expertly curated dataset of eye iriation and corrosion compounds, and validate multi-classification QSAR models to assess irritation potential of novel compounds	QSAR, RF, MuDRA	In Silico	• MuDRA models demonstrated high balanced accuracy (0.88, 0.85), sensitivity (0.89, 0.84), positive predictive value (0.90, 0.86), specificity (0.86, 0.86), and negative predictive value (0.85, 0.83) for irritation and corrosion respectively.
Tahsin 2022	Quasi-Experimental	Diabetes and Related Disorders	Rats	To evaluate the antidiabetic potential and safety profile of Gynura procumbens leaf extract through in vivo efficacy testing and in silico docking of phytocompounds against key glycemic enzymes.	Molecular docking	In Silico, In Vivo	• G. procumbens extract reduced blood glucose from 24.74 mmol/L to 18.05 mmol/L in diabetic rats ($p < 0.05$), comparable to metformin but without statistical superiority.<br>• 22 compounds from G. procumbens showed stronger binding affinities (up to –10.5 kcal/mol) to alpha-amylase and alpha-glucosidase than acarbose (standard reference, –7.6 kcal/mol).
Van Tongeren 2021	Diagnostic Test Accuracy	General Toxicity and Pharmacokinetic Prediction	None	To refine and validate the Dietary Comparator Ratio (DCR) framework for anti-androgenic risk assessment by defining new in vitro comparator exposure activity ratios (EARs) using the AR-	PBK Modeling	In Silico, In Vitro	• PBK-QIVIVE revealed that the predicted safe urinary DIM excretion was 84–533x higher than from 50g of Brussels sprouts.<br>• The safe BIC dose was ~4 orders of magnitude lower than therapeutic levels.

				CALUX assay and translating them to human-relevant exposures through PBK modeling-based QIVIVE.			
Wang 2024	Diagnostic Test Accuracy	Skin Sensitization	None	To develop reliable in silico methods for predicting skin sensitization using machine learning and the Dempster-Shafer theory (DST) to combine QSAR models.	QSAR Models, LR, RF, SVM, GDBT, ExtTree, XGBoost, LightGBM, MLP	In Silico	• Creation of a validated, interpretable in silico decision-support system (HSkinSensDS) capable of accurately predicting human skin sensitization, achieving up to 88% correct classification rate using selected evidence combinations.
Wang 2023	Quasi-Experimental	Neurological Disorders	Rats, Beagle Dogs	To develop a machine learning-based scoring function (RDFL) for identifying nonbioavailable substructures and to use this approach to design novel selective serotonin reuptake inhibitors (SSRIs) with improved oral bioavailability and CNS efficacy.	Molecular Docking, Relative Drug-Fragment Likelihood (RDFL) ML model	In Silico, In Vivo, In Vitro	• The RDFL model achieved 82.2% accuracy and AUC = 0.88 for the test set in classifying substructures by bioavailability.<br>• The redesigned compound DH4 showed significantly higher bioavailability (83.28% in rats) and 6.3-fold greater plasma exposure compared to vilazodone, while maintaining potent SERT inhibition and reducing depression-like behaviors in vivo.

Zhang 2024.	Diagnostic Test Accuracy	Infectious Diseases	Mice	To develop the first unified software package that uses artificial intelligence for high-throughput discovery and validation of novel antibacterial lysins from uncharacterized phage genomes.	ERT, LR, ANN, KNN, XGBoost, AlphaFold, Molecular Docking	In Silico, In Vivo, In Vitro	• The integrated method predicted hepatotoxicity with cross-validated AUC of 0.99.<br>• Predicted lysins were shown to have effective antimicrobial activity in vivo.
Zhou 2024	Diagnostic Test Accuracy	Ocular Toxicity	None	To develop robust computational tools to identify chemicals with potential ocular toxicity via machine-learning and deep-learning	RF, ADaBoost, ExtTree, XGBoost, LightGBM, SVM, GCN, AttentiveFP	In Silico	• Presentation of ML and DL models trained on the largest known ocular toxicity binary classification dataset achieved a peak AUC of 0.915.

References

Abbiati RA, Pourdehnad M, Carrancio S, Pierce DW, Kasibhatla S, McConnell M, Trotter MWB, Loos R, Santini CC, Ratushny AV. Quantitative Systems Pharmacology Modeling of Avadomide-Induced Neutropenia Enables Virtual Clinical Dose and Schedule Finding Studies. AAPS J. 2021 Aug 27;23(5):103. doi: 10.1208/s12248-021-00623-8.

Adnan M, Chy MNU, Kamal A, Chowdhury MR, Islam MS, Hossain MA, et al. Unveiling Pharmacological Responses and Potential Targets Insights of Identified Bioactive Constituents of Cuscuta reflexa Roxb. Leaves through In Vivo and In Silico Approaches. Pharmaceuticals (Basel). 2020;13(3)

Alam S, Rashid MA, Sarker MMR, Emon NU, Arman M, Mohamed IN, et al. Antidiarrheal, antimicrobial and antioxidant potentials of methanol extract of Colocasia gigantea Hook. f. leaves: evidenced from in vivo and in vitro studies along with computer-aided approaches. BMC Complement Med Ther. 2021;21(1):119

Alauddin M, Amin MR, Siddiquee MA, Hiwatashi K, Shimakage A, Takahashi S, et al. In silico and in vivo experiment of soymilk peptide (tetrapeptide - FFYY) for the treatment of hypertension. Peptides. 2024;175:171170

Albrecht W, Kappenberg F, Brecklinghaus T, Stoeber R, Marchan R, Zhang M, et al. Prediction of human drug-induced liver injury (DILI) in relation to oral doses and blood concentrations. Arch Toxicol. 2019;93(6):1609-37

Alves VM, Borba JVB, Braga RC, Korn DR, Kleinstreuer N, Causey K, et al. PreS/MD: Predictor of Sensitization Hazard for Chemical Substances Released From Medical Devices. Toxicol Sci. 2022;189(2):250-9.

Ambe K, Ishihara K, Ochibe T, Ohya K, Tamura S, Inoue K, et al. In Silico Prediction of Chemical-Induced Hepatocellular Hypertrophy Using Molecular Descriptors. Toxicol Sci. 2018;162(2):667-75

Bercu J, Masuda-Herrera MJ, Trejo-Martin A, Hasselgren C, Lord J, Graham J, et al. A cross-industry collaboration to assess if acute oral toxicity (Q)SAR models are fit-for-purpose for GHS classification and labelling. Regul Toxicol Pharmacol. 2021;120:104843

Chan JCY, Tan SPF, Upton Z, Chan ECY. Bottom-up physiologically-based biokinetic modelling as an alternative to animal testing. ALTEX. 2019;36(4):597-612

Chen C, Lavezzi SM, Iavarone L. The area under the effect curve as an efficacy determinant for anti-infectives. CPT Pharmacometrics Syst Pharmacol. 2022;11(8):1029-44

Chen X, Roberts R, Liu Z, Tong W. A generative adversarial network model alternative to animal studies for clinical pathology assessment. Nat Commun. 2023;14(1):7141

Di P, Zheng M, Yang T, Chen G, Ren J, Li X, et al. Prediction of serious eye damage or eye irritation potential of compounds via consensus labelling models and active learning models based on uncertainty strategies. Food Chem Toxicol. 2022;169:113420

Di Stefano M, Galati S, Piazza L, Granchi C, Mancini S, Fratini F, et al. VenomPred 2.0: A Novel In Silico Platform for an Extended and Human Interpretable Toxicological Profiling of Small Molecules. J Chem Inf Model. 2024;64(7):2275-89

Feng H, Zhang L, Li S, Liu L, Yang T, Yang P, et al. Predicting the reproductive toxicity of chemicals using ensemble learning methods and molecular fingerprints. Toxicol Lett. 2021;340:4-14

Gardiner LJ, Carrieri AP, Wilshaw J, Checkley S, Pyzer-Knapp EO, Krishna R. Using human in vitro transcriptome analysis to build trustworthy machine learning models for prediction of animal drug toxicity. Sci Rep. 2020;10(1):9522.

Garduno-Felix KG, Rochin-Medina JJ, Murua-Lopez CC, Lopez-Moreno HS, Ramirez K. Biostimulated-sesame sprout extracts as potential agents against Leishmania mexicana. Lett Appl Microbiol. 2023;76(10)

Gomatam A, Coutinho E. A chirality-sensitive approach to predict chemical transfer across the human placental barrier. Toxicol Lett. 2024;394:66-75.

Hartout P, Pocuca B, Mendez-Garcia C, Schleberger C. Investigating the human and nonobese diabetic mouse MHC class II immunopeptidome using protein language modeling. Bioinformatics. 2023;39(8)

Hassan A, Ikram A, Raza A, Saeed S, Zafar Paracha R, Younas Z, et al. Therapeutic Potential of Novel Mastoparan-Chitosan Nanoconstructs Against Clinical MDR Acinetobacter baumannii: In silico, in vitro and in vivo Studies. Int J Nanomedicine. 2021;16:3755-73

Im JE, Lee JD, Kim HY, Kim HR, Seo DW, Kim KB. Prediction of skin sensitization using machine learning. Toxicol In Vitro. 2023;93:105690.

Johnson C, Ahlberg E, Anger LT, Beilke L, Benigni R, Bercu J, et al. Skin sensitization in silico protocol. Regul Toxicol Pharmacol. 2020;116:104688

Kale MD, Kadam SP, Shravage BV, Nikam VS. From computational prediction to experimental validation: Hesperidin's anti-Urolithiatic activity in sodium oxalate-induced urolithiasis models in fruit flies and mice. Toxicol Appl Pharmacol. 2024;492:117104

Kamiya Y, Omura A, Hayasaka R, Saito R, Sano I, Handa K, et al. Prediction of permeability across intestinal cell monolayers for 219 disparate chemicals using in vitro experimental coefficients in a pH gradient system and in silico analyses by trivariate linear regressions and machine learning. Biochem Pharmacol. 2021;192:114749

Kammala AK, Richardson LS, Radnaa E, Han A, Menon R. Microfluidic technology and simulation models in studying pharmacokinetics during pregnancy. Front Pharmacol. 2023;14:1241815

Kim J, Yoon T, Kim P, Bekhbat M, Kang SM, Rho HS, et al. Manufactured tissue-to-tissue barrier chip for modeling the human blood-brain barrier and regulation of cellular trafficking. Lab Chip. 2023;23(13):2990-3001

Lawal B, Sani S, Onikanni AS, Ibrahim YO, Agboola AR, Lukman HY, et al. Preclinical anti-inflammatory and antioxidant effects of Azanza garckeana in STZ-induced glycemic-impaired rats, and pharmacoinformatics of it major phytoconstituents. Biomed Pharmacother. 2022;152:113196

Leedale J, Sharkey KJ, Colley HE, Norton AM, Peeney D, Mason CL, et al. A Combined In Vitro/In Silico Approach to Identifying Off-Target Receptor Toxicity. iScience. 2018;4:84-96

Leite DMC, Brochet X, Resch G, Que YA, Neves A, Pena-Reyes C. Computational prediction of inter-species relationships through omics data analysis and machine learning. BMC Bioinformatics. 2018;19(Suppl 14):420

Li T, Roberts R, Liu Z, Tong W. TransOrGAN: An Artificial Intelligence Mapping of Rat Transcriptomic Profiles between Organs, Ages, and Sexes. Chem Res Toxicol. 2023;36(6):916-25

Luo G, Lin X, Vega-Medina A, Xiao M, Li G, Wei H, et al. Targeting of the FOXM1 Oncoprotein by E3 Ligase-Assisted Degradation. J Med Chem. 2021;64(23):17098-114

Mohanty IR, Borde M, Kumar CS, Maheshwari U. Dipeptidyl peptidase IV Inhibitory activity of Terminalia arjuna attributes to its cardioprotective effects in experimental diabetes: In silico, in vitro and in vivo analyses. Phytomedicine. 2019;57:158-65

Paul K, Chakraborty S, Mallick P, Bhattacharjee P, Pal TK, Chatterjee N, et al. Supercritical carbon dioxide extracts of small cardamom and yellow mustard seeds have fasting hypoglycaemic effects: diabetic rat, predictive iHOMA2 models and molecular docking study. Br J Nutr. 2021;125(4):377-88

Peindl M, Gottlich C, Crouch S, Hoff N, Luttgens T, Schmitt F, et al. EMT, Stemness, and Drug Resistance in Biological Context: A 3D Tumor Tissue/In Silico Platform for

Analysis of Combinatorial Treatment in NSCLC with Aggressive KRAS-Biomarker Signatures. Cancers (Basel). 2022;14(9)

Price E, Gesquiere AJ. Animal simulations facilitate smart drug design through prediction of nanomaterial transport to individual tissue cells. Sci Adv. 2020;6(4):eaax2642.

Rani A, Sharma PB, Bhatia S, Sharma AK. Comprehensive study on pharmacognostic, pharmacological, and toxicological features of Ficus racemosa in Alzheimer's disease using GC-MS and molecular docking analyses. Toxicol Res (Camb). 2024;13(4):tfae098.

Russo G, Crispino E, Casati S, Corsini E, Worth A, Pappalardo F. Pioneering bioinformatics with agent-based modelling: an innovative protocol to accurately forecast skin or respiratory allergic reactions to chemical sensitizers. Brief Bioinform. 2024;25(6)

Shah A, Shah AA, Nandakumar K, Kumar A, Pai A, Lobo R. In-silico and in-vivo evaluation of sesamol and its derivatives for benign prostatic hypertrophy. 3 Biotech. 2021;11(9):411

Sharma B, Chenthamarakshan V, Dhurandhar A, Pereira S, Hendler JA, Dordick JS, et al. Accurate clinical toxicity prediction using multi-task deep neural nets and contrastive molecular explanations. Sci Rep. 2023;13(1):4908.

Silva AC, Borba J, Alves VM, Hall SUS, Furnham N, Kleinstreuer N, et al. Novel computational models offer alternatives to animal testing for assessing eye irritation and corrosion potential of chemicals. Artif Intell Life Sci. 2021;1

Tahsin MR, Tithi TI, Mim SR, Haque E, Sultana A, Bahar NB, et al. In Vivo and In Silico Assessment of Diabetes Ameliorating Potentiality and Safety Profile of Gynura procumbens Leaves. Evid Based Complement Alternat Med. 2022;2022:9095504

Van Tongeren TCA, Moxon TE, Dent MP, Li H, Carmichael PL, Rietjens I. Next generation risk assessment of human exposure to anti-androgens using newly defined comparator compound values. Toxicol In Vitro. 2021;73:105132.

Wang H, Huang Z, Lou S, Li W, Liu G, Tang Y. In Silico Prediction of Skin Sensitization for Compounds via Flexible Evidence Combination Based on Machine Learning and Dempster-Shafer Theory. Chem Res Toxicol. 2024;37(6):894-909

Wang ZZ, Yi C, Huang JJ, Xu TF, Chen KZ, Wang ZS, et al. Deciphering Nonbioavailable Substructures Improves the Bioavailability of Antidepressants by Serotonin Transporter. J Med Chem. 2023;66(1):371-83

Zhang Y, Li R, Zou G, Guo Y, Wu R, Zhou Y, et al. Discovery of Antimicrobial Lysins from the "Dark Matter" of Uncharacterized Phages Using Artificial Intelligence. Adv Sci (Weinh). 2024;11(32):e2404049

Zhou Y, Wang Z, Huang Z, Li W, Chen Y, Yu X, et al. In silico prediction of ocular toxicity of compounds using explainable machine learning and deep learning approaches. J Appl Toxicol. 2024;44(6):892-907
